# Protocol for SARS-CoV-2 infection of kidney organoids derived from human pluripotent stem cells

**DOI:** 10.1016/j.xpro.2022.101872

**Published:** 2022-11-07

**Authors:** Elena Garreta, Daniel Moya-Rull, Megan L. Stanifer, Vanessa Monteil, Patricia Prado, Andrés Marco, Carolina Tarantino, Maria Gallo, Gustav Jonsson, Astrid Hagelkruys, Ali Mirazimi, Steeve Boulant, Josef M. Penninger, Nuria Montserrat

**Affiliations:** 1 Pluripotency for Organ Regeneration, Institute for Bioengineering of Catalonia (IBEC), The Barcelona Institute of Science and Technology (BIST), 08028 Barcelona, Spain; 2 Department of Infectious Diseases, Molecular Virology, Heidelberg University Hospital, 69120 Heidelberg, Germany; 3 Research Group “Cellular Polarity and Viral Infection”, German Cancer Research Center (DKFZ), 69120 Heidelberg, Germany; 4 Department of Molecular Genetics and Microbiology, College of Medicine, University of Florida, Gainesville, FL, USA; 5 Karolinska Institute and Karolinska University Hospital, Unit of Clinical Microbiology, 17182 Stockholm, Sweden; 6 IMBA, Institute of Molecular Biotechnology of the Austrian Academy of Sciences, Dr. Bohr-Gasse 3, 1030 Vienna, Austria; 7 National Veterinary Institute, 751 89 Uppsala, Sweden; 8 Department of Infectious Diseases, Virology, Heidelberg University Hospital, Heidelberg, Germany; 9 Department of Medical Genetics, Life Sciences Institute, University of British Columbia, Vancouver, BC V6T 1Z3, Canada; 10 Catalan Institution for Research and Advanced Studies (ICREA), Barcelona, Spain; 11 Centro de Investigación Biomédica en Red en Bioingeniería, Biomateriales y Nanomedicina, 28029 Madrid, Spain

**Keywords:** Cell culture, Microbiology, Microscopy, Stem Cells, Cell Differentiation, Organoids

## Abstract

This protocol presents the use of SARS-CoV-2 isolates to infect human kidney organoids, enabling exploration of the impact of SARS-CoV-2 infection in a human multicellular *in vitro* system. We detail steps to generate kidney organoids from human pluripotent stem cells (hPSCs) and emulate a diabetic milieu via organoids exposure to diabetogenic-like cell culture conditions. We further describe preparation and titration steps of SARS-CoV-2 virus stocks, their subsequent use to infect the kidney organoids, and assessment of the infection via immunofluorescence.

For complete details on the use and execution of this protocol, please refer to Garreta et al. (2022).^1^

## Highlights

• Description of technical details to generate kidney organoids from hPSCs
• Approach to induce diabetic-like hallmarks in kidney organoids
• Methodology to prepare and titrate SARS-CoV-2 virus stocks and infect kidney organoids
• Evaluate viral infection in whole organoids or paraffin sections by immunofluorescence

## Abstract (editor highlights)

Publisher’s note: Undertaking any experimental protocol requires adherence to local institutional guidelines for laboratory safety and ethics.

## Graphical abstract

**Figure:**
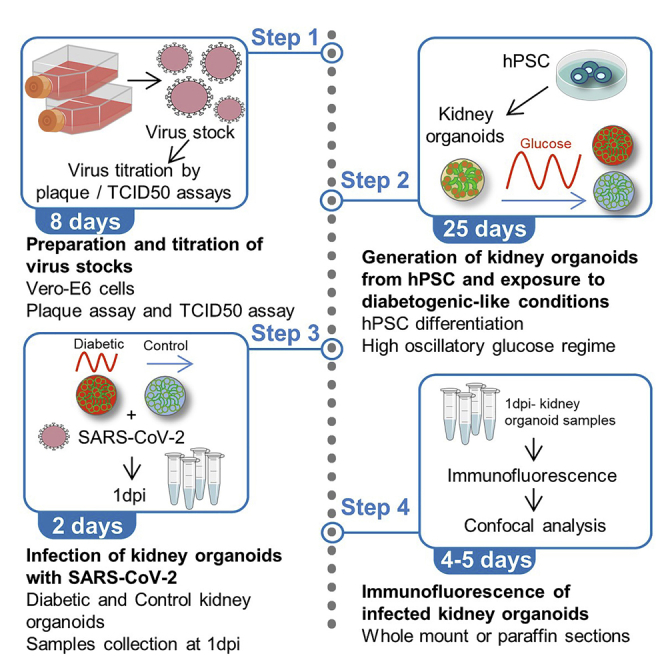

## Before you begin

The protocol herein outlines the detailed procedures for using SARS-CoV-2 virus isolates to infect human kidney organoids. Specifically, we describe the steps to prepare SARS-CoV-2 virus stocks and determine virus titers using Vero-E6 Cells. We also detail the methodology to culture and differentiate hPSC into kidney organoids, and how to induce diabetic-like kidney organoids *in vitro*. Next, we explain how to efficiently infect human kidney organoids with SARS-CoV-2 and assess infection by immunofluorescence and confocal imaging.

**CRITICAL:** All laboratory procedures related to virus preparation, infection of cell cultures/organoids or collection of infected cells/organoids must be performed in certified class II biological safety cabinets in a Biosafety Level 3 (BSL-3) containment laboratory. Personnel are required to wear appropriate personal protective equipment to protect from biological risks as determined by local requirements. Personal equipment as well as virus handling and decontamination of all materials must be performed before exiting the containment area. This includes decontamination of all tubes/samples that exit the BSL-3 area as well as ensuring that all protocols to collect RNA and protein are shown to kill any infectious virus. The protocols that state whether a virus has been inactivated must be tested, reviewed and approved by the competent Institutional Biosafety Committees or National agencies depending on the requirements at your institution.

### Institutional permissions

SARS-CoV-2 can be isolated from patient samples with proper institutional approval. Additionally, SARS-CoV-2 variants can be obtained through international banks after confirmation that you are certified to have the proper BSL-3 containment to receive and propagate the virus. All approvals are Institute specific, and you should verify the local regulations prior to starting any infectious disease work. All experiments handling SARS-CoV-2 virus i.e., (but not limited to) isolation, culture, infection and inactivation must be separately reviewed and validated by the competent Institutional Biosafety Committees before the beginning of the project.

### Culture of Vero-E6 cells

**Timing: 1 week**

This procedure describes the culture and expansion of Vero-E6 Cells and can be performed in BSL-1 or BSL-2 containment.

1. Supplement Dulbecco’s Modified Eagle’s Medium (DMEM) with 1% Minimum Essential Medium Non-Essential Amino Acids (MEM NEAA), 10 mM HEPES Buffer Solution and 10% Fetal Bovine Serum (FBS) (Complete Growth Medium).
2. Pre-warm Complete Growth Medium at 37°C.
3. Thaw a cryovial with Vero-E6 Cells in a 37°C water bath.
  a. Check the cryovial every 20 s. As soon as the cryovial content becomes liquid, transfer the cell suspension using a p1000 micropipette to a sterile 15 mL conical tube with 10 mL of pre-warmed Complete Growth Medium.
  b. Centrifuge the cell suspension at 300 × *g* for 5 min.
  c. Remove the supernatant and re-suspend the cell pellet in 10 mL pre-warmed Complete Growth Medium and carefully pipette up and down to create a homogeneous solution.
  d. Count cells.
    i. Mix thoroughly 10 μL of cell suspension and 10 μL of 0.4% Trypan Blue Stain, and pipette 10 μL of the mixture into a Countess^TM^ cell counting chamber slide.
      ***Note:*** Trypan Blue is used to discriminate live from dead cells. Dead cells will be stained blue and should not be counted.
    ii. Count viable cells using a Countess Automated Cell Counter.
      ***Alternatives:*** A Neubauer cell counting chamber can be used to count viable cells under the microscope.

4. Dilute the cell suspension in Complete Growth Medium to have a cell density between 1.5–3.5 × 10^5^ viable cells/mL. Then, plate 15 mL of the cell suspension in a 75-cm^2^ flask (3 × 10^4^ to 7 × 10^4^ cells/cm^2^).

***Note:*** Vero-E6 Cells recovery improves by plating cells at high densities post-thaw (3 × 10^4^ to 7 × 10^4^ cells/cm^2^). Small 25-cm^2^ or 50-cm^2^ cell culture flasks can also be used for this purpose. For a 25-cm^2^ flask, re-suspend the cells in 5 mL of Complete Growth Medium; for a 50-cm^2^ flask, re-suspend the cells in 10 mL of Complete Growth Medium.

5. Incubate flasks at 37°C and 5% CO_2_.
6. Split the cells when 80%–90% confluency is achieved using a split ratio of 1:10 (the dilution ratio depends on the cells needed for subsequent experiments. Use a split ratio 1:4 if you need cells 2–3 days after splitting. Use 1:10 if you need cells 7 days later or for on-going culture).
  a. Remove medium and rinse cells with Phosphate Buffered Saline (PBS) 1×.
  b. Remove the PBS 1× and add 1 mL (25-cm^2^ flask) or 3 mL (75-cm^2^ flask) of 0.25% Trypsin-Ethylenediaminetetraacetic acid (EDTA) solution.
  c. Allow the flask to sit at 37°C to ensure cell detachment. This usually takes 3–5 min.
  d. Quench the Trypsin-EDTA by adding an equal volume of fresh Complete Growth Medium.
  e. Collect and pipette up and down to create a homogeneous solution and plate into new culture flasks.
  f. Top up flask with extra Complete Growth Medium to reach a final volume of 10 mL for a 25-cm^2^ or 15 mL for a 75-cm^2^ flask.

***Note:*** After recovery from frozen stock, Vero-E6 Cells need 2–3 passages to reach their regular growth rate.

***Note:*** Prepare frozen stocks of Vero-E6 Cells from early passages. A confluent 75-cm^2^ flask can be used to make 5 cryovials. To freeze cells, following trypsinization of a 75-cm^2^ flask (see steps 6a–e above), add media back to cells to reach a total volume of 10 mL. Pellet cells by centrifugation at 300 × *g* for 5 min. Remove the supernatant and add 5 mL of freezing medium consisting of FBS supplemented with 10% Dimethyl Sulfoxide (DMSO) to re-suspend the cell pellet. Transfer 1 mL of the cell suspension using a p1000 micropipette into each labelled cryovial. Immediately place the cryovials with the cells into a Mr Frosty™ Freezing Container and store at −80°C for 24 h before to stock them in liquid nitrogen.

### Culture of human pluripotent stem cells

**Timing: 2 weeks**

This procedure describes the culture and expansion of hPSC using Complete Essential 8 (E8) Medium and Vitronectin (VTN-N) coated culture plates. Culture and expansion of hPSC should be performed in BSL-2 containment.

7. Prepare Complete E8 Medium.
  a. Thaw E8 Supplement (50×) at 4°C for 12–16 h. Do not thaw at 37°C.
  b. Add 10 mL of E8 Supplement and 5 mL of 10.000 U/mL Penicillin/Streptomycin (P/S) into 485 mL of E8 Basal Medium to obtain 500 mL of Complete E8 Medium.

***Note:*** Complete E8 Medium can be stored at 2°C–8°C for up to 2 weeks. Before use, warm only the required amount of medium at 20°C–22°C. Do not warm the medium at 37°C.

8. Prepare VTN-N coated 6-well plates.
  a. Thaw the VTN-N vial at 4°C. To avoid multiple freezing and thawing cycles prepare 60 μL VTN-N aliquots in sterile 1.5 mL Eppendorf tubes and freeze them at –80°C.
    ***Note:*** 60 μL of VTN-N is the amount required to coat one 6-well plate.
  b. Dilute VTN-N 1:100 in Dulbecco’s PBS (DPBS) 1× to have a VTN-N solution at 0.5 μg/mL.
    ***Note:*** 60 μL of VTN-N is diluted in 6 mL of DPBS 1×.
  c. Place 1 mL of the VTN-N solution into each well of 6-well plate.
  d. Incubate at 20°C–22°C for 1 h.
  e. Before plating cells, aspirate the VTN-N solution from each well.
    ***Note:*** VTN-N coated plates can be stored at 4°C for up to a week. Store the plates with the VTN-N solution. Do not allow drying. Prior to use, pre-warm the culture plate at 20°C–22°C for 1 h. Before plating cells, aspirate the VTN-N solution from each well.
    ***Note:*** Other plate formats can be used to maintain and expand hPSC although 6 well-pates are recommended.

9. Prepare 0.5 mM EDTA in DPBS 1×.
  a. Dilute 50 μL of 0.5 M EDTA in 50 mL of DPBS 1×.
  b. Filter the obtained solution using a 0.22 μm pore size syringe filter.

***Note:*** The 0.5 mM EDTA solution can be stored at 20°C–22°C for up to 6 months.

10. Thaw a cryovial containing hPSC in a 37°C water bath. Troubleshooting 1.
  a. After carefully submerging the cryovial in the water bath check the cryovial content every 20 s. As soon as the cryovial content becomes liquid, transfer the cell suspension using a p1000 micropipette to a sterile 15 mL conical tube containing 10 mL of Complete E8 Medium.
  b. Centrifuge the cell suspension at 300 × *g* for 5 min. Remove the supernatant and gently re-suspend the cell pellet in 2 mL of Complete E8 Medium to obtain a homogeneous cell suspension.
  c. Count cells.
    i. Take 50 μL of cell suspension in a 1.5 mL Eppendorf tube.
    ii. Add 150 μL of Accumax and incubate at 37°C for 5 min to obtain a single cell suspension.
    iii. Mix 10 μL of the single cell suspension with 10 μL of 0.4% Trypan Blue Stain and pipette 10 μL of the mixture into a Countess^TM^ cell counting chamber slide.
    iv. Count viable cells using a Countess Automated Cell Counter.
    v. Consider that the initial cell suspension is diluted 1:4 with Accumax. Thus, the final number of cells per mL is [Number of viable cells] × 4.

***Alternatives:*** A Neubauer cell counting chamber can be used to count viable cells under the microscope.

11. Dilute the cells into the volume of Complete E8 Medium required for achieving a cell density between 4.75–5.7 × 10^5^ viable cells/mL. Then, plate 2 mL of the cell suspension per well of VTN-N coated 6-well plate (1–1.2 × 10^5^ cells/cm^2^).

**CRITICAL:** When thawing hPSC, do not add ROCK inhibitor to the medium. hPSC routinely exposed to ROCK inhibitor may lead to inefficient generation of kidney organoids.

**CRITICAL:** After thawing, hPSC should be passaged at least twice before using the cells for subsequent differentiation methodologies.

12. Incubate the 6-well plate containing hPSC at 37°C and 5% CO_2_. Refresh with Complete E8 Medium the day after cell plating. Change medium every other day.

13. Routinely, passage hPSC when the colonies cover 80% of the surface area of the culture plate, usually every 4–6 days (see Figure 1). Troubleshooting 2 and 3.
  a. Aspirate the Complete E8 Medium from each well of 6-well plate.
  b. Wash with 2 mL of DPBS 1× per well.
  c. Add 1 mL of 0.5 mM EDTA per well and incubate for 4–6 min at 20°C–22°C. During incubation with EDTA, observe the hPSC colonies under the microscope. When the cells in the colonies start to round up and detach, they are ready to be removed from the well.
    **CRITICAL:** Avoid longer EDTA exposure. Excessive cell dissociation may result in low cell viability.
  d. Aspirate the EDTA solution from the wells without disrupting the hPSC colonies. Then, dissociate the colonies by flushing them with 1 mL of Complete E8 Medium (2–3 times) to break up the colonies into small cell clusters.
    **CRITICAL:** Do not pipette the cell clusters more than 3 times, because hPSC are sensitive to mechanical stress. Excessive mechanical disruption would result in low cell viability.
  e. Re-suspend the cell clusters in Complete E8 Medium and plate 2 mL of the cell suspension per well in a new VTN-N coated 6-well plate. Use a split ratio ranging from 1:3 to 1:4 for the first 2 passages post-thaw and from 1:6 to 1:10 for subsequent passages.
    **CRITICAL:** Do not add ROCK inhibitor to the medium during hPSC passage.
    ***Note:*** The optimal split ratio and dilution ratio during a split may vary among different hPSC lines and will need to be adjusted to allow growth of the cell colonies for 4–6 days until reaching 80% confluency.
  f. Incubate the 6-well plate containing hPSC at 37°C and 5% CO_2_. Refresh with 2 mL of Complete E8 Medium per well the day after cell plating. Change medium every other day.

**Figure 1:**
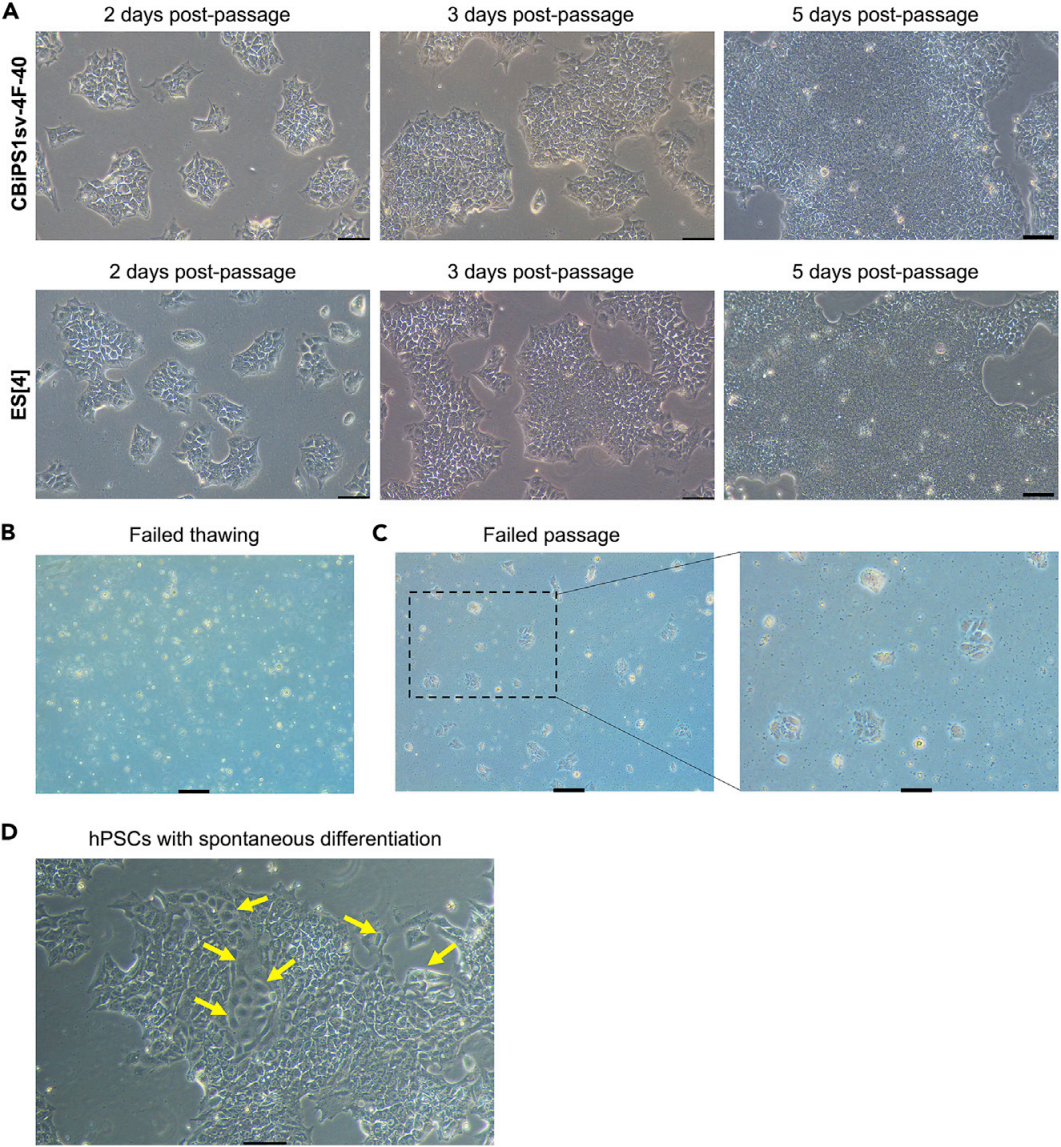
Typical morphology of hPSC colonies cultured on VTN-N-coated plates with E8 medium (A) Representative bright field images of the hiPSC line CBiPS1sv-4F-40 and the hESC line ES[4] at day 2, day 3 and day 5 after passaging. After 5 days in culture, hPSC colonies show nearly 80% confluency. (B) Representative bright field image taken 24 h after thawing an hPSC cryovial that shows dead floating hPSCs because of a failed thawing procedure. (C) Representative bright field image taken 48 h after hPSC passaging that shows very few small cell clusters indicating a failed passage procedure. (D) Representative bright field images of hPSC colonies with spontaneous differentiation. Yellow arrows indicate differentiated cells. Scale bars in (A-D), 100 μm.
14. Prepare frozen stocks of hPSC.
  a. Prepare Freezing Medium containing 10% DMSO by mixing 9 mL of Complete E8 Medium with 1 mL of DMSO in a sterile 15 mL conical tube.
  b. When hPSC colonies cover 80% of the surface area of the culture plate, dissociate them from each well of 6-well plate into small cell clusters using 0.5 mM EDTA as detailed in steps 13a–d.
    ***Note:*** Usually freeze 1 well of 6-well plate of 80% confluent hPSC per cryovial.
  c. Centrifuge the cell suspension at 300 × *g* for 5 min. Remove the supernatant. For each well of dissociated hPSC, use 1 mL of ice-cold Freezing Medium to gently re-suspend the cell pellet.
  d. Transfer 1 mL of the cell suspension using a p1000 micropipette into each labelled cryovial. Immediately place the cryovials with the cells into a Mr Frosty™ Freezing Container and store at −80°C for 24 h.
  e. The day after, transfer the frozen cryovials from −80°C freezer to a nitrogen tank.

## Key resources table

**Table:** 

REAGENT or RESOURCE	SOURCE	IDENTIFIER
**Antibodies**		

Human/mouse/rat/hamster angiotensin-converting enzyme 2 (ACE2) antibody, Source: Polyclonal Goat IgG, Working dilution: 1:20	Bio-Techne R&D Systems	Cat# AF933; RRID: AB_355722
SARS-CoV/SARS-CoV-2 nucleocapsid antibody for virus nuclear protein (NP) detection, Source: Monoclonal Mouse IgG1 Clone #05, Working dilution: 1:1000 (for immunofluorescence in Vero-E6 cells), 1:500 (for immunofluorescence in kidney organoids)	Abyntek Biopharma	Cat# 40143-MM05; RRID: AB_2827977
IRDye® 800CW Goat anti-Mouse IgG Secondary Antibody, Working dilution: 1:5000	Licor	Cat# 926-32210; RRID: AB_621842
Alexa Fluor® 488 AffiniPure Donkey Anti-Goat IgG (H+L), Working dilution: 1:200	Jackson ImmunoResearch	Cat# 705-545-147; RRID: AB_2336933
Cy™3 AffiniPure Donkey Anti-Mouse IgG (H+L), Working dilution: 1:200	Jackson ImmunoResearch	Cat# 715-165-151; RRID: AB_2315777
Streptavidin, DyLight^TM^ 649 Conjugated, Working dilution: 1:40	Vector Labs	Cat# SA-5649; RRID: AB_2336421

**Bacterial and virus strains**		

SARS-CoV-2, GENBANK: MT093571	Isolated from patient; Monteil et al.^2^ Garreta et al.^1^	N/A

**Chemicals, peptides, and recombinant proteins**		

Lotus Tetragonolobus Lectin (LTL), biotinylated, Working dilution: 1:200	Vector Laboratories	Cat# B-1325; RRID: AB_2336558
Draq5	Thermo Fisher	Cat# 62254
4′,6-Diamidino-2-phenylindole, dihydrochloride (DAPI)	Thermo Fisher	Cat# D1306
Essential 8 (E8) Medium consisting of Essential 8™ Basal Medium and Essential 8™ Supplement (50×)	Thermo Fisher	Cat# A1517001
Vitronectin (VTN-N), Recombinant Human Protein, Truncated	Thermo Fisher	Cat# A14700
Trypan Blue stain (0.4%)	Thermo Fisher	Cat# 15250061
Ethylenediaminetetraacetic acid (EDTA) solution	Thermo Fisher	Cat# 15575038
ACCUMAX™ Cell detachment solution	Stem Cell Technologies	Cat# 07921
0.25% Trypsin-EDTA solution (1×)	Thermo Fisher	Cat# 25200056
TrypLE™ Express	Thermo Fisher	Cat# 12604021
Advanced RPMI 1640	Thermo Fisher	Cat# 12633020
Minimum Essential Medium Non-Essential Amino Acids (MEM NEAA) (100×)	Thermo Fisher	Cat# 11140035
HEPES (1 M) buffer solution	Thermo Fisher	Cat# 15630080
GlutaMAX™ Supplement (200 mM)	Thermo Fisher	Cat# 35050038
Penicillin/streptomycin (P/S) (10.000 U/mL)	Thermo Fisher	Cat# 15140122
Fetal bovine serum (FBS)	Thermo Fisher	Cat# 10270106
Dulbecco’s Modified Eagle Medium (DMEM)	Thermo Fisher	Cat# 11995065
DMEM, glucose-free	Thermo Fisher	Cat# 11966025
Minimum Essential Medium – Eagle with Earle’s BSS (EMEM) (2×)	Lonza	Cat# BE12-668F
D-Glucose	Merck	Cat# G7021
D-Mannitol	Merck	Cat# M9647
CHIR99021 (CHIR)	Merck	Cat# SML1046; CAS: 2 52917-06-9
Recombinant human FGF9	PeproTech	Cat# 100-23
Heparin	Merck	Cat# H3149; CAS: 9041-08-1
Activin A	R&D Systems	Cat# 338-AC-050
Dulbecco’s phosphate buffered saline (DPBS) without calcium and magnesium (1×)	Thermo Fisher	Cat# 14190144
Distilled water, cell culture grade	Thermo Fisher	Cat# 15230089
Phosphate buffered saline (PBS) pH 7.4 (1×)	Thermo Fisher	Cat# 1001015
Fluoromount-G	Southern Biotech	Cat# 0100-01
RapiClear® 1.47	Sunjin Lab	Cat# RC147001
Triton X-100	Sigma	Cat# T8787
Dimethyl sulfoxide (DMSO)	Merck	Cat# D2650; CAS: 67-68-5
Formaldehyde solution (min. 37%)	Sigma-Aldrich	Cat# 1039991000
Paraformaldehyde (PFA)	Aname	Cat# sc-281692
Donkey serum (DS)	Sigma-Aldrich	Cat# S30-100ML
Bovine serum albumin (BSA)	Sigma-Aldrich	Cat# A4503
Trizma base	Sigma-Aldrich	Cat# T6791
Sodium chloride (NaCl)	Sigma-Aldrich	Cat# S7653
Sodium azide	Sigma-Aldrich	Cat# 71290
Low gelling temperature type VII Agarose	Sigma-Aldrich	Cat# A4018
SeaKem ME Agarose	Lonza	Cat# 50014
Crystal Violet	Thermo Fisher	Cat# C581-100
Ethanol absolute (99.8%)	PanReac AppliChem	Cat# PANR.141086.1211; CAS: 64-17-5
Hydrochloric Acid (HCl), 1 mol/L (1 N) volumetric solution	PanReac AppliChem	Cat# 181021.1211 CAS: 7647-01-0

**Critical commercial assays**		

Streptavidin/Biotin Blocking Kit	Vector Laboratories	Cat# SP-2002
Intercept® (PBS) Blocking Buffer	Licor	Cat# 927-70001

**Experimental models: Cell lines**		

ES[4] Human Embryonic Stem Cell line	The National Bank of Stem Cells (ISCIII, Madrid)	https://www.isciii.es/
CBiPS1sv-4F-40 Human Induced Pluripotent Stem Cell line	The National Bank of Stem Cells (ISCIII, Madrid)	https://www.isciii.es/
Vero-E6	ATCC	Cat# ATCC CRL-1586

**Software and algorithms**		

Empiria Studio® Software	Licor	N/A
Fiji ImageJ2 version 2.3.0	NIH	https://imagej.net/software/fiji/

**Other**		

Countess™ Cell Counting Chamber Slides	Thermo Fisher	Cat# C10228
Falcon® 15 mL Polystyrene Centrifuge Tube, Conical Bottom, with Dome Seal Screw Cap, Sterile	Corning	Cat# 352099
Falcon® 50 mL High Clarity PP Centrifuge Tube, Conical Bottom, Sterile	Corning	Cat# 352070
Screw Cap Micro Tube, working volume: 2 mL, skirted conical base, Sterile	Sarstedt	Cat# 72.694.005
Eppendorf™ Safe-Lock Tubes, 1.5 mL	Fisher Scientific	Cat# 10509691
Mr Frosty™ Freezing Container	Thermo Fisher	Cat# 5100-0001
Fisherbrand™ Internally Threaded Cryogenic Storage Vials, 2 mL, Sterile	Fisher Scientific	Cat# 11321675
Merck Millex™-GP Sterile Syringe Filter Units with PES Membrane, 0.22 μm	Fisher Scientific	Cat# 16437555
Nunc™ EasYFlasks™ TC-treated with Filter Cap, 75-cm^2^ flask	Thermo Fisher	Cat# 156499
Nunc™ 96-Well Polystyrene Conical Bottom (V-bottom) MicroWell™ Plates	Thermo Fisher	Cat# 249935
96 Well Black/Clear Bottom Plate, TC Surface	Thermo Fisher	Cat# 165305
96 Well Plate, Sphera Low-Attachment Surface	Thermo Fisher	Cat# 174927
200 μL Graduated Tip/Wide Orifice, Racked	Ecogen	Cat# E1011-8400
Histology plastic molds	Laboquimia	Cat# 20447200820
Charged glass slides SUPERFROST Plus	VWR	Cat# 631-9483
Menzel-Gläser Coverslip 24 × 50 mm #1.5, Rectangular coverslips, 24 × 50 mm, #1.5	VWR	Cat# 631-9430
iSpacer®	Sunjin Lab	Cat# IS211

## Materials and equipment

### Preparation of medium for Vero-E6 cell culture and infection with SARS-CoV-2

**Table:** Complete Growth Medium

Reagent	Final concentration	Volume (mL)
DMEM (High Glucose)	N/A	435
P/S (10.000 U/mL)	1%	5
MEM NEAA 100×	1%	5
FBS	10%	50
HEPES (1 M)	10 mM	5
**Total**	**N/A**	**500**

Store at 4°C, use within 6 weeks, bring to 37°C prior to use.

### Preparation of reagents and solutions for the plaque assay and TCID50

**Table:** Complete Infection Medium

Reagent	Final concentration	Volume (mL)
DMEM (High Glucose)	N/A	485
P/S (10.000 U/mL)	1%	5
FBS	2%	10
**Total**	**N/A**	**500**

Store at 4°C, use within 6 weeks, bring to 37°C prior to use.

**Table:** Agarose solution (1%)

Reagent	Final concentration	Amount
SeaKem ME Agarose	1%	1 g
Deionized distilled (Milli-Q) water	N/A	100 mL
**Total**	**1%**	**100 mL**

Dissolve 1g of Agarose in 100 mL of boiling Milli-Q water. Autoclave. Keep at 20°C–22°C.

Before to use, melt the agarose using a microwave and keep at 55°C in a water-bath until use.

**Table:** EMEM/14% FBS Medium

Reagent	Final concentration	Volume (mL)
EMEM 2×	N/A	42
FBS	14%	7
GlutaMAX (200 mM)	2%	1
**Total**	**N/A**	**50**

Store at 4°C, use within 6 weeks, bring to 20°C–22°C prior to use.

**Table:** Plaque Assay Overlay Medium

Reagent	Final concentration	Volume (mL)
EMEM/14% FBS Medium	N/A	50
Agarose solution (1%)	0.5%	50
**Total**	**N/A**	**100**

Equilibrate the EMEM+14% FBS at 20°C–22°C. Prepare the plaque assay overlay medium just before adding the overlay on cells by mixing at 1:1 dilution ratio EMEM+14%FBS (at 20°C–22°C) and 1% agarose solution (at 55°C). Use immediately to overlay the cell monolayers.

**Table:** Formaldehyde solution (25%)

Reagent	Final concentration	Volume (mL)
Formaldehyde (37%)	25%	33.8
PBS 1×	N/A	16.2
**Total**	**25%**	**50 mL**

Store at 4°C, use within 1 week. Formaldehyde solutions must be disposed of safely and in accordance with the local regulations.

**Table:** Crystal Violet staining solution (1% w/v)

Reagent	Final concentration	Amount
Crystal violet	1%	1 g
Absolute Ethanol	20%	20 mL
Formaldehyde (37%)	25%	25 mL
PBS 1×	N/A	55 mL
**Total**	**1%**	**100 mL**

Store at 20°C–22°C protected from light, use within 4 months. Mix before each use.

### Preparation of medium for hPSC maintenance

**Table:** Complete E8 Medium

Reagent	Final concentration	Volume (mL)
E8 Basal Medium	N/A	485
E8 Supplement 50×	N/A	10
P/S (10.000 U/mL)	1%	5
**Total**	**N/A**	**500**

Store at 4°C, use within 2 weeks. Before use, warm only the required amount of medium at 20°C–22°C. Do not warm the medium at 37°C. Protect from direct light.

### Stock preparation of medium components for kidney organoid generation

**Table:** Stock solution of CHIR

Reagent	Final concentration (mM)	Amount
CHIR	12	5 mg
DMSO	N/A	896 μL
**Total**	**12**	**896** μL

Make 50 μL working aliquots and store at −20°C, use within 6 months.

**Table:** Stock solution of Activin A (50 μg/mL)

Reagent	Final concentration (μg/mL)	Amount
Activin A	50	50 μg
Sterile HCl (4 mM)	N/A	1,000 μL
**Total**	**50**	**1,000** μL

Make 20–50 μL working aliquots and store at −20°C, use within 6 months. Avoid repeated freezing and thawing cycles.

**Table:** Stock solution of Recombinant Human FGF9 (50 μg/mL)

Reagent	Final concentration (μg/mL)	Amount
FGF9	50	500 μg
Distilled Water, Cell culture grade	N/A	10,000 μL
**Total**	**50**	**10,000** μL

Make 100 μL working aliquots and store at −20°C, use within 6 months. Avoid repeated freezing and thawing cycles.

**Table:** Stock solution of Heparin (50 mg/mL)

Reagent	Final concentration (mg/mL)	Amount
Heparin	50	50 mg
Distilled Water, Cell culture grade	N/A	1,000 μL
**Total**	**50**	**1,000** μL

Make 50 μL aliquots and store at 4°C, use within 2 years. For regular use, prepare an intermediate heparin stock solution at 5 mg/mL by diluting the 50 mg/mL stock at 1:10 ratio.

### Preparation of medium for kidney organoid generation

**Table:** Complete Advanced RPMI 1640 Medium

Reagent	Final concentration (%)	Volume (mL)
Advanced RPMI 1640 Basal Medium	N/A	49
P/S (10.000 U/mL)	1	0.5
GlutaMAX (200 mM)	1	0.5
**Total**	**N/A**	**50**

Store at 4°C, use within 4 weeks, bring to 20°C–22°C prior to use.

**Table:** Posterior Primitive Streak Induction Medium

Reagent	Final concentration	Volume
Complete Advanced RPMI 1640 Medium	N/A	15 mL
CHIR (12 mM)	8 μM	10 μL
**Total**	**N/A**	**15 mL**

Prepare fresh, bring to 20°C–22°C prior to use.

**Table:** Intermediate Mesoderm Induction Medium

Reagent	Final concentration	Volume
Complete Advanced RPMI 1640 Medium	N/A	15 mL
FGF9 (50 μg/mL)	200 ng/mL	60 μL
Activin A (50 μg/mL)	10 ng/mL	3 μL
Heparin (5 mg/mL)	1 μg/mL	3 μL
**Total**	**N/A**	**15 mL**

Prepare fresh, bring to 20°C–22°C prior to use. For heparin, use an intermediate stock solution at 5 mg/mL by diluting the 50 mg/mL stock at 1:10 ratio.

**Table:** CHIR-Pulse Organoid Induction Medium

Reagent	Final concentration	Volume
Complete Advanced RPMI 1640 Medium	N/A	15 mL
FGF9 (50 μg/mL)	200 ng/mL	60 μL
Heparin (5 mg/mL)	1 μg/mL	3 μL
CHIR (12 mM)	5 μM	6.25 μL
**Total**	**N/A**	**15 mL**

Prepare fresh, bring to 20°C–22°C prior to use. For heparin, use an intermediate stock solution at 5 mg/mL by diluting the 50 mg/mL stock at 1:10 ratio.

**Table:** Kidney Organoid Differentiation Medium

Reagent	Final concentration	Volume
Complete Advanced RPMI 1640 Medium	N/A	40 mL
FGF9 (50 μg/mL)	200 ng/mL	160 μL
Heparin (5 mg/mL)	1 μg/mL	8 μL
**Total**	**N/A**	**40 mL**

Prepare fresh, bring to 20°C–22°C prior to use. For heparin, use an intermediate stock solution at 5 mg/mL by diluting the 50 mg/mL stock at 1:10 ratio.

### Stock preparation of medium components and complete medium for high oscillatory glucose treatment of kidney organoids

**Table:** Stock solution of D-Glucose (1 M)

Reagent	Final concentration	Amount
D-Glucose	1 M	18.0 g
Distilled Water, Cell culture grade	N/A	100 mL
**Total**	**1 M**	**100 mL**

Mix 18 g of glucose with 80 mL of cell culture grade distilled water using magnetic stirring until complete dissolution. Then, bring to a total volume of 100 mL with cell culture grade distilled water. Filter the solution using a 0.22 μm pore size syringe filter and store at 4°C, use within 2 months. Do not autoclave glucose solutions.

**Table:** Stock solution of D-Mannitol (1 M)

Reagent	Final concentration	Amount
D-Mannitol	1 M	18.2 g
Distilled Water, Cell culture grade	N/A	100 mL
**Total**	**1 M**	**100 mL**

Mix 18.2 g of mannitol with 80 mL of cell culture grade distilled water using magnetic stirring until complete dissolution. Then, bring to a total volume of 100 mL with cell culture grade distilled water. Filter the solution using a 0.22 μm pore size syringe filter. Store at 20°C–22°C to avoid crystal formation, use within 2 months.

**Table:** Normoglycemic Medium (5 mM Glucose)

Reagent	Final concentration	Volume (mL)
DMEM (Glucose free)	N/A	477.5
P/S (10.000 U/mL)	1%	5
GlutaMAX (200 mM)	1%	5
D-Glucose (1 M)	5 mM	2.5
D-Mannitol (1 M)	20 mM	10
**Total**	**N/A**	**500**

Store at 4°C, use within 6 weeks, bring to 37°C prior to use.

**Table:** Hyperglycemic Medium (25 mM Glucose)

Reagent	Final concentration	Volume (mL)
DMEM (glucose free)	N/A	477.5
P/S (10.000 U/mL)	1%	5
GlutaMAX (200 mM)	1%	5
D-Glucose (1 M)	25 mM	12.5
**Total**	**N/A**	**500**

Store at 4°C, use within 6 weeks, bring to 37°C prior to use.

### Preparation of solutions for immunofluorescence analysis of kidney organoids

**Table:** PFA fixative solution (4%)

Reagent	Final concentration	Volume (mL)
PFA (16%)	4%	12.5
PBS 1×	N/A	37.5
**Total**	**4%**	**50**

Store at 4°C, use within 1 week. PFA solutions must be disposed of safely and in accordance with the local regulations.

**Table:** Tris-Buffered Saline (TBS) 10× (pH 7.6)

Reagent	Final concentration	Amount
Trizma base	200 mM	24 g
NaCl	1.5 M	88 g
Distilled (Milli-Q) water	N/A	900 mL
**Total**	**N/A**	**1,000 mL**

Dissolve 24 g of Trizma base and 88 gr NaCl in 900 mL of distilled (Milli-Q) water. Then adjust pH to 7.6 with hydrochloric acid (HCl), and complete with distilled (Milli-Q) water until 1,000 mL final volume. Store at 4°C, use within 6 months.

**Table:** TBS 1× (pH 7.6)

Reagent	Final concentration	Volume (mL)
TBS 10×	N/A	100
Distilled (Milli-Q) water	N/A	900
**Total**	**N/A**	**1,000**

Store at 4°C, use within 6 months.

**Table:** TBS 1× supplemented with 1% Triton X-100

Reagent	Final concentration	Volume (mL)
TBS 1×	N/A	100
Triton 100-X	1%	1
**Total**	**N/A**	**100**

Store at 4°C, use within 2 weeks.

**Table:** TBS 1× supplemented with 0.5% Triton X-100

Reagent	Final concentration	Volume (mL)
TBS 1×	N/A	100
Triton 100-X	0.5%	0.5
**Total**	**N/A**	**100**

Store at 4°C, use within 2 weeks.

**Table:** Blocking buffer for whole mount immunofluorescence

Reagent	Final concentration	Volume
TBS 1× supplemented with 1% Triton X-100	N/A	9.4 mL
Donkey serum	6%	600 μL
**Total**	**N/A**	**10 mL**

Filter the solution using a 0.22 μm pore size syringe filter and store at 4°C, use within 1 week.

**Table:** Blocking buffer for immunofluorescence in paraffin sections

Reagent	Final concentration	Volume
TBS 1× supplemented with 1% Triton X-100	N/A	9.7 mL
Donkey Serum	3%	300 μL
**Total**	**N/A**	**1,000**

Filter the solution using a 0.22 μm pore size syringe filter and store at 4°C, use within 1 week.

**Table:** TBS 1× supplemented with 1% Triton X-100 and 1% BSA

Reagent	Final concentration	Amount
TBS 1× supplemented with 1% Triton X-100	N/A	50 mL
BSA	1%	0.5 g
**Total**	**N/A**	**50 mL**

Filter the solution using a 0.22 μm pore size syringe filter and store at 4°C, use within 1 week.

**Table:** TBS 1× supplemented with 0.5% Triton X-100 and 1% BSA

Reagent	Final concentration	Amount
TBS 1× supplemented with 0.5% Triton X-100	N/A	50 mL
BSA	1%	0.5 g
**Total**	**N/A**	**50 mL**

Filter the solution using a 0.22 μm pore size syringe filter and store at 4°C, use within 1 week.

**Table:** Primary antibody dilution ratios for whole mount or paraffin section immunofluorescence

Primary antibody or reagent	Dilution ratio
ACE2	1:20
NP	1:500
LTL	1:200

Dilute the primary antibodies at the indicated dilution ratios in TBS 1× supplemented with 1% Triton X-100 and 1% BSA for whole mount immunofluorescence or in TBS 1× supplemented with 0.5% triton X-100 and 1% BSA for organoid paraffin sections. Prepare fresh.

**Table:** Secondary antibody dilution ratios for whole mount or paraffin section immunofluorescence

Secondary antibody or reagent	Dilution ratio
Anti-Goat-AF488	1:200
Anti-mouse-Cy3	1:200
Streptavidin- Dylight 649	1:40

Dilute the secondary antibodies at the indicated dilution ratios in TBS 1× supplemented with 1% Triton X-100 and 1% BSA for whole mount immunofluorescence or in TBS 1× supplemented with 0.5% Triton X-100 and 1% BSA for organoid paraffin sections. Prepare fresh.

**Table:** Stock of DAPI (5 mg/mL)

Reagent	Final concentration	Amount
DAPI	5 mg/mL (14.3 mM)	10 mg
Distilled (Milli-Q) water	N/A	2 mL
**Total**	**5 mg/mL (14.3 mM)**	**2 mL**

Make 20–50 μL aliquots and store at −20°C in the dark, use within 6 months. Avoid repeated freezing and thawing cycles.

**Table:** DAPI working solution (1:5000)

Reagent	Final concentration	Volume
DAPI (5 mg/mL)	1 μg/mL	2 μL
TBS 1×	N/A	10 mL
**Total**	**N/A**	**10 mL**

Prepare fresh. DAPI solutions must be disposed of safely and in accordance with the local regulations.

## Step-by-step method details

### Preparation of SARS-CoV-2 virus stocks

**Timing: 4 days**

This step allows the production of virus stock by amplification of a defined SARS-CoV-2 virus strain in Vero-E6 Cells. The infectivity of virus stock is assessed by 2 different means including plaque assay (next section, beginning at step 7) or by tissue culture infectious dose-50 (TCID50) assay (beginning at step 14).

1. Grow Vero-E6 Cells until 90% confluency in two 75-cm^2^ flasks.
  a. 24 h before virus inoculation, plate Vero-E6 Cells at 6.5–9.5 × 10^4^ cells/cm^2^ in ten 75-cm^2^ flasks in Complete Growth Medium. Each 75-cm^2^ flask will produce 15 mL of virus stock.
  b. Plate 2 additional flasks, 1 for mock-infected control and the other one for cell counting.
    ***Note:*** It is desirable to produce large amount of virus at a time to have consistency between experimental replicates. Usually, 10 flasks of Vero-E6 Cells for SARS-CoV-2 infection can be prepared. Vero-E6 Cells need to be 85%–90% confluent for virus inoculation and propagation.
  c. Incubate the flasks at 37°C and 5% CO_2_.
    **CRITICAL:** Restart the culture with a new early passage of Vero-E6 Cells if changes in morphology or if virus titers have decreased to below 10^5^ plaque-forming units (PFU)/mL.
    **CRITICAL:** SARS-CoV-2 should not be passaged more than 3 times in Vero-E6 Cells to avoid viral genome mutations that may decrease or change its infectivity.

2. The day after seeding, count the cells and calculate the volume of virus inoculum to have a multiplicity of infection (MOI) of 0.01 PFU per cell using the following formula:

Volume of virus needed in mL= (Number of cells in the flask) × (desired MOI) / (virus inoculum titer in PFU/mL).

***Note:*** A 90% confluent 75-cm^2^ flask contains approximately 6.5–8 × 10^6^ cells. For a virus titer of 10^7^ PFU/mL, the volume of virus needed to inoculate 1 75-cm^2^ flask of Vero-E6 Cells would be: (6.5 × 10^6^) × (0.01) / 10^7^ = 0.0065 mL= 6.5 μL per flask.

**CRITICAL:** To produce SARS-CoV-2 virus stocks, it is important to inoculate the virus at low MOI (MOI=0.01 per cell) to ensure that each cell is productively infected while preventing the formation of defective viral particles.

**CRITICAL:** Steps 1–2 can be performed in BSL-1 or BSL-2 containment. From this step on, all procedures (steps 3–6) must occur under BSL-3 containment.

**Figure 2:**
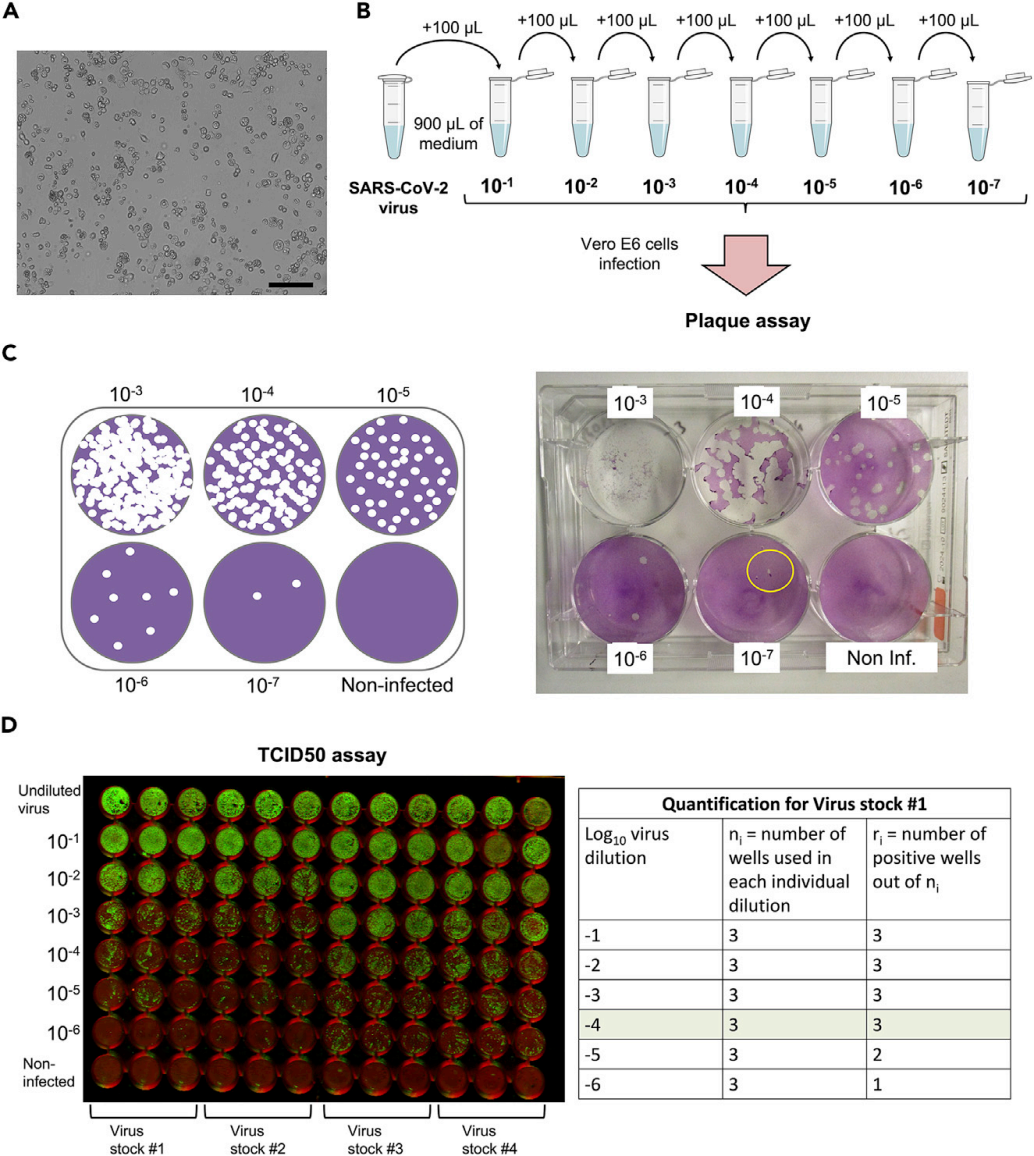
Propagation and titration of SARS-CoV-2 virus in Vero-E6 Cells (A) Representative bright field image of Vero-E6 Cell monolayer with apparent CPE 72 h after SARS-CoV-2 virus inoculation. Scale bar, 100 μm. (B) Schematics of the virus serial dilutions preparation. (C) Schematics of a 6-well plate used for the plaque assay after staining with the crystal violet solution (left) and the corresponding image of a real plaque assay plate (right) showing the formation of the plaques. Note that the well corresponding to the 10^-7^ virus dilution does not show plaque formation but presents a scratch (indicated with a yellow circle) that has been accidentally performed when removing the agarose layer with the flat spoon and can be confounded with a plaque. (D) Representative fluorescent image of a typical 96-well plate prepared for virus titer assessment by the TCID50 assay (left). The table (right) shows a representative quantification of the number of positive infected wells that are used to calculate the virus titer by the Spearman-Karber method.
6. When 90% CPE is observed, harvest the virus supernatant from infected Vero-E6 Cell monolayers.
  a. Collect the virus supernatant into sterile 15 mL conical tubes. Discard the mock-infected control.
    i. Dispose flasks, remaining cells and pipettes as infectious waste.
  b. Centrifuge the tubes at 450 × *g* for 5 min at 4°C to pellet the cell debris.
  c. Prepare 200–500 μL aliquots of the supernatant in labelled 2 mL screw-tubes.
  d. Immediately store the aliquots of virus stock at −80°C.

***Note:*** The volume of virus stock aliquots will need to be adjusted to avoid repeated freezing and thawing cycles. This will prevent decrease of virus stock infectivity.

3. Thaw a vial of SARS-CoV-2 seed stock at 20°C–22°C.
4. Dilute the necessary volume of virus (calculated above) in DMEM supplemented with 2% FBS (Complete Infection Medium).
5. Inoculate 90% confluent Vero-E6 Cell monolayers with virus.
  a. Remove the medium from the 75-cm^2^ flasks containing 90% confluent Vero-E6 Cells.
  b. Wash once with 3 mL of PBS 1× per flask.
  c. Add 3 mL of Complete Infection Medium containing the virus (from step 4) to each 75-cm^2^ flask. For mock-infected control, add 3 mL of fresh Complete Infection Medium.
  d. Incubate flasks at 37°C and 5% CO_2_ for 1 h.
  e. After 1 h, add 7 mL of Complete Growth Medium to each flask.
  f. Monitor the flasks daily for 48–72 h for the appearance of cytopathic effect (CPE) using an inverted microscope (see Figure 2A). Compare with mock-infected flask as negative control.

### Titration of SARS-CoV-2 virus stock by plaque assay

**Timing: 4 days**

This step is required to determine the virus stock titer by plaque assay, which measures infectious SARS-CoV-2 particles by quantifying the plaques generated in Vero-E6 Cell cultures after infection with serial dilutions of the virus stock using a viscous medium overlay. The viscous overlay is used to restrict the spreading of progeny virions to the initially infected foci of cells. In this manner, each infectious particle will generate a single area of cellular death, so called PFU. After incubation, cell monolayers are fixed and stained to visualize the plaques using crystal violet, which stains cells in purple while plaques remain clear. Plaques are then counted to assess the virus titer in PFU per mL (PFU/mL).

7. Prepare Vero-E6 Cell monolayers for plaque assay.
  a. Plate 8 × 10^5^ Vero-E6 Cells in 3 mL of Complete Growth Medium per well of 6-well plate.
    ***Note:*** One 6-well plate is prepared per virus stock to be tested.
  b. Incubate the plate for 12–16 h at 37°C and 5% CO_2_.
  c. After 12–16 h incubation, check under the microscope that cells formed a confluent compact monolayer in each well.
    **CRITICAL:** Step 7 can be performed in BSL-1 or BSL-2 containment. From this step on, all procedures (steps 8–13) must occur under BSL-3 containment.

8. Heat the 1% Agarose solution in a microwave and keep the bottle in a water-bath at 55°C.
9. Prepare EMEM/14% FBS Medium and equilibrate it at 20°C–22°C.
10. Dilute the virus stock (see Figure 2B).
  a. Prepare 10-fold serial dilutions of the virus sample between 10^-1^ to 10^-7^.
    i. For each virus sample to be titrated, take seven 1.5 mL Eppendorf tubes and label them with each dilution (10^-1^, 10^-2^, 10^-3^, 10^-4^, 10^-5^, 10^-6^, 10^-7^).
    ii. Add 900 μL of Complete Infection Medium to each tube.
    iii. Add 100 μL of the virus sample to be titrated to the first tube (10^-1^) to make the first dilution.
    iv. Mix by vortexing the tube. This is your 10^-1^ virus dilution.
    v. Continue with serial dilutions by taking 100 μL of the first virus dilution (10^-1^) and transferring it to the second tube (10^-2^).
    vi. Mix by vortexing the tube. This is now your 10^-2^ virus dilution.
    vii. For subsequent virus dilutions repeat steps v-vi by taking 100 μL of the newly created virus dilution and adding it to the next Eppendorf tube until you have reached a 10^-7^ dilution.

**CRITICAL:** Directly discard the tips without pipetting between each dilution.

11. Infect Vero-E6 Cell monolayers prepared in step 7 with the serial virus dilutions.
  a. Label each well of 6-well plate with the corresponding dilution ranging from 10^-3^ to 10^-7^ and 1 well for non-infected control.
  b. Remove the existent Complete Growth Medium from the Vero-E6 Cell monolayers.
  c. Wash once with 1 mL of PBS 1× per well.
  d. After vortexing the tube, transfer 500 μL of the 10^-7^ virus dilution to one well of the labelled 6-well plate.
  e. Repeat this step for 10^-6^ to 10^-3^ dilutions.
    ***Note:*** It is recommendable to start with the most diluted sample so the same pipette tip can be then used for subsequent pipetting from the most diluted to the less diluted samples.
  f. Incubate the plates at 37°C and 5% CO_2_ for 1 h to allow virus to attach.
    **CRITICAL:** Redistribute the supernatant by rocking the plates carefully every 15 min to prevent cell monolayers from drying out.
  g. During the incubation time, calculate the volume of Plaque Assay Overlay Medium needed, counting 3 mL per well of 6-well plate. Include 3 mL extra as a control of polymerization.
    ***Note:*** For example, for a 6-well plate, 6 × 3 mL + 3 mL are needed, i.e 21 mL of plaque assay overlay medium.
  h. After 1 h incubation, remove the viral inoculums from each well and close the plate.
    **CRITICAL:** Change tips between each well.
  i. Prepare the Plaque Assay Overlay Medium in a 50 mL conical tube, by quickly mixing 1 volume of 1% Agarose solution (equilibrated at 55°C, see step 8) and 1 volume of EMEM/14% FBS Medium (equilibrated at 20°C–22°C, see step 9) (1:1 ratio).
    **CRITICAL:** The overlay medium has to be prepared just before being distributed in the plate because agarose begins to polymerize rapidly after mixing with the EMEM/14% FBS Medium.
  j. Add 3 mL of Plaque Assay Overlay Medium to each well of 6-well plate.
    **CRITICAL:** Let the overlay polymerize in the plate under the hood. To check the polymerization status, check the status of the leftover in the 50 mL tube.
  k. Incubate the plates at 37°C and 5% CO_2_ for 3 days to allow plaque formation.

12. Visualize plaques by Crystal Violet staining 3 days post-infection (see Figure 2C). Troubleshooting 4 and 5.
  ***Note:*** Crystal Violet stains proteins and DNA within intact cells. In this manner, areas of infected dead cells will appear as clear spots on the purple cell monolayer.
  a. Add 1 mL of 25% Formaldehyde solution per well on top of the agarose to allow fixation of the cell monolayers.
  b. Incubate for 30 min at 20°C–22°C.
    ***Note:*** The fixation step with 25% formaldehyde solution allows for virus inactivation.
  c. Carefully remove the agarose layer using a flat spatula. Dispose as infectious waste.
    **CRITICAL:** Do not touch the cell monolayer with the spatula to avoid scratching the monolayer.
  d. Add 1 mL of 1% Crystal Violet staining solution per well of the 6-well plate.
  e. Incubate for 10 min at 20°C–22°C.
  f. Remove the Crystal Violet solution from the wells and wash 3 times with 3 mL of tap water per well.
    **CRITICAL:** Properly discard formaldehyde and crystal violet solutions as toxic waste, according to institutional rules.
  g. Leave the plate inverted to air-dry the stained monolayers.
    **Pause point:** At this point, air-dried stained plates can be stored at 20°C–22°C for further quantification.

13. Enumerate plaques and calculate the virus titer. Troubleshooting 6 and 7.
  a. Count the plaques in each well at each virus dilution.
    ***Note:*** Discount wells with fewer than 5 or greater than 100 plaques. Take note of plaque size and morphology. The negative control should present a uniform, purple-stained monolayer and is used as a reference control.
  b. Identify the first dilution in which a maximum of 50 plaques can be counted. Determine the viral titer of the virus using the following formula:

Plaque forming units (PFU)/mL = [average No. of plaques] / [Dilution factor × Volume of inoculum added in mL].

***Note:*** For instance, if you counted 50 plaques in the well corresponding to 10^-5^ dilution in which you have added 0.5 mL of diluted virus inoculum, the titer of the virus stock would be 50/ (10^-5^ × 0.5) = 10^7^ PFU/mL.

***Note:*** We recommend to run the titration in duplicate or to repeat the titration step using another tube from the same viral stock.

### Titration of SARS-CoV-2 virus stock by TCID50 assay

**Timing: 3 days**

As an alternative method to the plaque assay, this step details how to determine the virus stock titer by TCID50 assay.

14. Prepare Vero-E6 Cell monolayers in a 96-well Black/Clear Bottom plate (96-BCB plate).
  a. Plate 30,000 Vero-E6 Cells in 100 μL of Complete Growth Medium per well of a 96-BCB plate. Prepare enough wells to test each virus dilution in triplicate.
    ***Note:*** One 96-BCB plate can be used to test 4 different virus stocks in triplicate (see Figure 2D).
  b. Incubate the plate 12–16 h at 37°C and 5% CO_2_.
  c. After 12–16 h incubation, check under the microscope that cells have formed a confluent compact monolayer in each well.
    **CRITICAL:** Step 14 can be performed in a BSL-1 or BSL-2 containment. Steps 15–21 must occur under BSL-3 containment.

15. Infect the Vero-E6 Cells prepared in step 14 with 10-fold serial virus dilutions.
  a. Remove the 100 μL of media from the first row of wells of the 96-BCB plate containing Vero-E6 Cells.
  b. Add 110 μL of non-diluted virus stock per well.
  c. Use a multichannel pipette to remove 10 μL of virus from the first row of wells and place it in the second row of wells. Gently pipette up and down to mix the virus with the media. This is now your 10^-1^ virus dilution.
  d. Repeat step 15c by removing 10 μL of the virus dilution from the second row of wells (10^-1^) and adding it to the third row of wells. This is now your 10^-2^ virus dilution.
  e. For subsequent virus dilutions repeat step 15c by removing 10 μL of the newly created virus dilution and adding it to the next row of wells until you have reached a 10^-6^ dilution.
  f. Incubate the 96-BCB plate for 24 h at 37°C and 5% CO_2_.

**CRITICAL:** Change the pipette tips for each dilution and make sure to properly mix the contents of the well before diluting into the next well. Leave the last lane as a mock sample.

16. Fix the 96-BCB plate at 24 h post-infection.
  a. Remove the virus-infected media.
  b. Wash the wells 1 time with 200 μL of PBS 1× per well.
  c. Add 200 μL of 4% Paraformaldehyde (PFA) to each well and incubate at 20°C–22°C for 20 min.
  d. Remove the PFA and wash the wells 3 times with 200 μL of PBS 1× per well.

***Note:*** The fixation step with 4% PFA allows for virus inactivation.

**CRITICAL:** After the 20 min-fixation, the full plate should be submerged in 4% PFA solution and incubated for additional 20 min to ensure that any virus found in the plate is inactivated. Once completely inactivated, next steps can be performed in the BSL-1/BSL-2 laboratory.

17. Perform immunolabelling of the fixed 96-BCB plate to detect virus infection (see Figure 2D).
  a. Add 100 μL of PBS 1× supplemented with 0.05% Triton-X to each well. Incubate at 20°C–22°C for 15 min.
  b. Remove the solution and add 100 μL per well of the Intercept® (PBS) Blocking Buffer. Incubate at 20°C–22°C for 30 min.
  c. Prepare the necessary primary antibody solution by diluting the primary antibody against the virus nuclear protein (NP) at 1:1000 dilution ratio in Intercept® (PBS) Blocking Buffer.
  d. Incubate with the primary antibody solution as indicated below:
    i. Remove the Intercept® (PBS) Blocking Buffer from the wells and add 100 μL of the primary antibody solution to each well.
    ii. Incubate at 20°C–22°C for 1 h.
  e. Remove the primary antibody solution from the wells and wash 3 times with 100 μL of PBS 1× per well.
  f. Prepare the necessary secondary antibody solution by diluting the anti-mouse IR800CW secondary antibody at 1:5000 dilution ratio in Intercept® (PBS) Blocking Buffer. Supplement this solution also with the nuclear dye Draq5 at 1:10000 dilution ratio.
  g. Incubate with the secondary antibody solution as indicated below:
    i. Add 100 μL of the secondary antibody solution supplemented with the nuclear dye Draq5 to each well.
    ii. Incubate at 20°C–22°C in the dark for 45 min.
  h. Remove the secondary antibody solution and wash the wells 3 times with 100 μL of PBS 1× per well.
  i. Add 200 μL of PBS 1× to each well and image the plate in an Odyssey® Imaging System (Odyssey® M, CLx or DLx models by Licor). Analyse with the Empiria Studio® Software.

***Alternatives:*** An epifluorescence microscope can also be used instead of the Odyssey® Imaging System to image the immunolabelled plate. In this case, use PBS 1× supplemented with 5% BSA as blocking buffer and perform the secondary antibody incubation using a secondary antibody conjugated to Alexa Fluor 488 at 1:1000 dilution ratio and the nuclear stain DAPI at 1:2000 dilution ratio in blocking buffer.

18. Determine the number of positive infected wells using the following method:
  a. Determine the average intensity of mock-infected cells.
  b. Determine the standard deviation of the intensity of mock-infected cells.
  c. Multiple the standard deviation by 2.
  d. Add the 2× the standard deviation to the average mock value. All values above this are considered positive wells.

***Note:*** For example – Average Intensity=180.2, standard deviation=23.4, 2× standard deviation=46.8, therefore all wells which have an intensity over 180.2 + 46.8 = 227.1 are considered positive.

19. Use the Spearman-Karber method to calculate the TCID50/mL using the following formula:

log_10_ 50% end point dilution = - (x_0_ - d/2 + d ∑ r_i_/n_i_).

x_0_ = log_10_ of the reciprocal of the highest dilution (lowest concentration) at which all wells are positive.

d = log_10_ of the dilution factor (dilution factor= 10).

n_i_ = number of wells used in each individual dilution.

r_i_ = number of positive wells out of n_i_.

Summation is started at dilution x_0_.

***Note:*** For example, if the highest virus dilution at which all wells are positive is 10^-4^, then x_0_ = 4; d = 1; log_10_ of 50% endpoint dilution = - [4 - ½ + 1 ((3+2+1)/3)] = -5.5; Therefore, the 50% end point dilution= 10^-5.5^ and the titer of the virus = 10^5.5^ TCID50/mL (see Figure 2D).

20. TCID50/mL can be used to calculate the PFU/mL using the following conversion:

PFU/mL = TCID50 × 0.69.

***Note:*** For example, if TCID50/mL = 10^5.5^, PFU/mL = 10^5.5^ × 0.69 = 3.2 × 10^5^ × 0.69 = 2.2 × 10^5^ PFU/mL.

### Generation of kidney organoids from human pluripotent stem cells

**Timing: 18 days**

The following steps describe the generation of kidney organoids from hPSC in a 96-well floating culture system.^1^^,^^2^ This protocol is an adaptation of our previous methodology describing the generation of kidney organoids using the transwell culture system^3^ and our previous published works using 96-well floating culture system.^2^^,^^4^ In the next section, day 16 kidney organoids generated in floating culture conditions are exposed to normoglycemic-like glucose culture regimes or high oscillatory glucose culture conditions to emulate a diabetic-like milieu (beginning at step 26).

21. Seeding of hPSC for differentiation (day -2 to day 0), see Figure 3A. Troubleshooting 8.
  a. 1 h prior cell dissociation, prepare one 24-well plate with VTN-N coating (as described in before you begin section). Use 0.3 mL of VTN-N solution per well of 24-well plate.
    ***Note:*** A typical differentiation experiment is started from 1 VTN-N coated 24-well plate.
  b. Disaggregate 3 wells of 6-well plate with hPSC at 80% of confluency into small cell clusters, as it is performed for a normal hPSC passage (described in before you begin section).
    ***Note:*** A typical differentiation experiment can be initiated from 3 wells of 6-well plate with hPSCs at 80% of confluency. The number of cells that can be obtained from 1 well of 6-well plate with hPSC colonies at 80% of confluency normally ranges from 2 to 3 million cells.
  c. Collect the resultant cell clusters in 6 mL of Complete E8 Medium in a sterile 15 mL conical tube.
  d. Count cells using a Countess Automated Cell Counter (see before you begin section step 10c).
    ***Alternatives:*** A Neubauer cell counting chamber can be used to count viable cells under the microscope.
  e. Plate between 1–2 × 10^5^ viable cells per well of VTN-N coated 24-well plate.
    **CRITICAL:** The starting cell density and colony distribution is crucial for an efficient differentiation and can vary among different hPSC lines. Usually, the optimal cell density ranges between 1–2 × 10^5^ cells per well of 24-well plate and should be adjusted for every hPSC line.
    i. Calculate the volume of Complete E8 medium needed to achieve a cell density between 2–4 × 10^5^ cells/mL. Re-suspend the cells and plate 0.5 mL of the cell suspension per well of VTN-N coated 24-well plate.
    ii. After 24 h, refresh with Complete E8 Medium.
    iii. Incubate at 37°C and 5% CO_2_ for 24 h.

**Figure 3:**
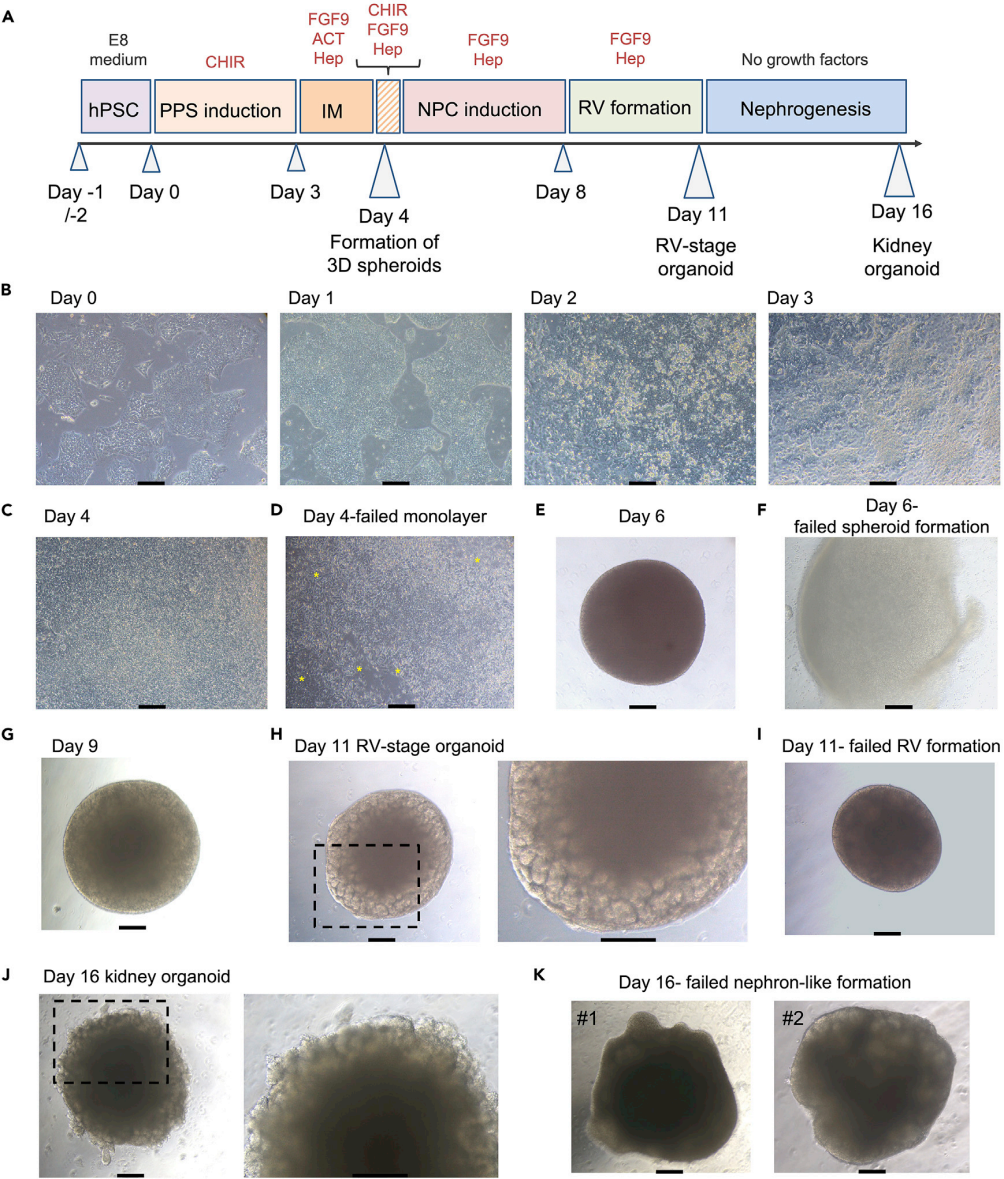
Generation of human kidney organoids from hPSC (A) Timeline of kidney organoid generation from hPSCs. PPS: posterior primitive streak; IM: intermediate mesoderm; NPC: nephron progenitor cell; RV: renal vesicle; ACT: activin A; Hep: heparin. (B) Representative bright field images showing morphological changes in the cell monolayer from day 0 to day 3 of differentiation. (C) Representative bright field image of the cell monolayer at day 4 of differentiation showing a very compact and uniform appearance. (D) Example of a failed formation of a day 4 cell monolayer. Yellow asterisks indicate areas of loose clusters of cells or empty areas. (E) Representative bright field image of a day 6 spheroid (2 days after intermediate mesoderm-committed cell aggregation into spheroids). At this stage, cell spheroid is characterized by a well-compacted round morphology. (F) Example of a failed cell aggregation in a day 6 spheroid. Note that edges are broken and disaggregated. (G) Representative bright field image of a day 9 organoid that begin to show the formation of RVs in the organoid edges. (H) Representative bright field image of a day 11 RV-stage organoid containing many RVs. (I) Example of a failed formation of RVs in a day 11 kidney organoid. (J) Representative bright field image of a day 16 kidney organoid containing multiple nephron-like structures. (K) Examples of inefficient formation of nephron-like structures within day 16 kidney organoids. Scale bars in (B–K), 100 μm.
22. Induction of posterior primitive streak formation (day 0 to day 3), see Figure 3A. Troubleshooting 9.
  a. Gently aspirate the Complete E8 medium from each well of the 24-well plate prepared in step 21 without disrupting the hPSC colonies (day 0).
  b. Add 0.5 mL of Complete Advanced RPMI 1640 Basal Medium supplemented with 8 μM CHIR (Posterior Primitive Streak Induction Medium) to each well. Incubate the plate at 37°C and 5% CO_2_.
  c. Replace the media with fresh Posterior Primitive Streak Induction Medium every 24 h for 3 consecutive days (see Figure 3B).
23. Induction of intermediate mesoderm formation (day 3 to day 4), see Figure 3A. Troubleshooting 10.
  a. Gently aspirate the Posterior Primitive Streak Induction Medium from each well of the 24-well plate avoiding to mechanically disrupt the cell monolayers (day 3).
  b. Add 0.5 mL per well of Complete Advanced RPMI 1640 Basal Medium supplemented with 200 ng/mL FGF9, 1 μg/mL Heparin and 10 ng/mL Activin A (Intermediate Mesoderm Induction Medium). Incubate the plate at 37°C and 5% CO_2_ for 24 h (see Figures 3C and 3D).

**CRITICAL:** It is key for the success of kidney organoid generation that the treatment with FGF9, Activin A and Heparin lasts exactly 24 h.

24. Generation of Kidney organoids (day 4 to day 16), see Figure 3A.
  **CRITICAL:** On day 4 of differentiation observe cells under a microscope and check for the presence of a tight homogeneous cell monolayer. If day 4 cell monolayers have not grown properly, showing large empty areas of cells, the organoid generation can be compromised.
  a. Gently aspirate the Intermediate Mesoderm Induction Medium from each well of the 24-well plate avoiding to mechanically disrupt the cell monolayers (day 4).
  b. Add 0.5 mL of Complete Advanced RPMI 1640 basal medium supplemented with 5 μM CHIR, 200 ng/mL FGF9 and 1 μg/mL Heparin (CHIR-Pulse Organoid Induction Medium). Incubate the plate at 37°C and 5% CO_2_ for 1 h.
  c. After 1 h incubation, dissociate cell monolayers to generate a single cell suspension.
    **CRITICAL:** At this point, cell monolayers are fragile and can break easily. Pay special attention during media changes and washing steps to avoid losing cells.
    i. Gently aspirate the supernatant without mechanically disrupting the cell monolayer.
    ii. Rinse cell monolayers by adding 0.5 mL of PBS 1× per well of 24-well plate.
    iii. Add 300 μL of TrypLE™ Express and incubate at 20°C–22°C for 1 min.
    iv. Gently aspirate the TrypLE™ Express without mechanically disrupting the cell monolayer.
    v. Dissociate the cell monolayers by flushing 0.5 mL of Complete Advanced RPMI 1640 Basal Medium supplemented with 200 ng/mL FGF9 and 1 μg/mL Heparin (Kidney Organoid Differentiation Medium) to each well of 24-well plate.
    vi. Collect the resultant cell suspension in a 50 mL conical tube. Mix 10 μL of cell suspension and 10 μL of 0.4% Trypan Blue Stain, mix thoroughly and pipette 10 μL of the mixture into a Countess^TM^ cell counting chamber slide. Count viable cells using a Countess Automated Cell Counter or a Neubauer chamber.
      ***Note:*** The average number of day 4-intermediate mesoderm committed cells that are obtained out of 1 well of 24-well plate ranges from 1.5–3 × 10^6^ cells.
  d. Plate 1 × 10^5^ cells per well of Nunc™ 96-Well Polystyrene Conical Bottom (V-bottom) MicroWell™ plate.
    i. Centrifuge the cell suspension at 300 × *g* for 5 min and re-suspend the cells in Kidney Organoid Differentiation Medium at a cell density of 1 × 10^6^ cells/mL.
    ii. Use a 12-multichannel pipette to plate 100 μL of the resultant cell suspension per well of V-bottom MicroWell™ plate.
  e. Centrifuge V-bottom MicroWell™ plates at 300 × *g* for 3 min to induce the formation of intermediate mesoderm-committed spheroids (1 single organoid per well will form). Troubleshooting 11.
  f. Incubate the resultant organoids at 37°C and 5% CO_2_ (see Figures 3E and 3F).
  g. Culture the organoids for 7 additional days (day 4 to day 11) in Kidney Organoid Differentiation Medium. Change medium every second day.
    i. Use a 12-multichannel pipette to remove the media avoiding touching the organoids and to add 100 μL of fresh Kidney Organoid Differentiation Medium per well.
      **CRITICAL:** When changing media of V-bottom MicroWell™ plates with organoids pay attention not to aspirate organoids by accident.
    ii. Incubate the plate at 37°C and 5% CO_2_.
  h. On day 11 of differentiation, remove growth factors by culturing organoids in 100 μL per well of Complete Advanced RPMI 1640 Medium. Organoids with numerous renal vesicles (RV) should be visible by day 11 (see Figures 3G–3I). Troubleshooting 12.
    ***Note:*** Discard the experiment if RV have not appeared by day 12.
  i. Culture the developing kidney organoids for 5 additional days (day 11 to day 16) in Complete Advanced RPMI 1640 Medium. Change medium every second day.
    ***Optional:*** From day 11 of differentiation, media changes can be performed every third day by adding 140 μL of Complete Advanced RPMI 1640 medium per well.

25. On day 16 of differentiation, kidney organoids show multiple nephron-like structures (see Figures 3J and 3K). Troubleshooting 13.

### Challenging kidney organoids with high oscillatory glucose to emulate a diabetic-like milieu *in vitro*

**Timing: 7 days**

This section details the steps required to treat kidney organoids (generated in steps 21–25 of previous section) with normoglycemic or high oscillatory glucose culture regimes.^1^ Organoids cultured under both experimental conditions are infected with SARS-Cov2 as described in the next section (step 28).

26. Maintain organoids in V-bottom MicroWell™ plate and expose them to normoglycemic (control) or high oscillatory glucose (diabetic) conditions for 7 days (from day 16 to day 23).
  a. Use a 12-multichannel pipette to replace the media every day with 100 μL per well of Normoglycemic or Hyperglycemic Medium. Incubate at 37°C and 5% CO_2_.
  b. Control organoids are cultured in Normoglycemic Medium only.
  c. Diabetic organoids are exposed to Normoglycemic Medium on even days or Hyperglycemic Medium on odd days.

**CRITICAL:** When performing medium changes ensure to completely replace the media from each well to guarantee the proper exposure of kidney organoids to the desired glucose concentration. Pay attention not to aspirate organoids by accident.

27. After the 7 day-treatment, use control and diabetic kidney organoids for SARS-CoV-2 infection experiments (beginning at step 28).

### Infection of kidney organoids with SARS-CoV-2

**Timing: 2 days**

This step describes the methodology to infect control or diabetic kidney organoids with SARS-CoV-2. Mock and SARS-CoV-2 infected kidney organoids are analysed to detect the viral RNA by qRT-PCR and the virus nuclear protein (NP) by immunofluorescence. For RNA extraction, qRT-PCR analysis and primer sequences, please refer to Garreta et al. (2022).^1^ For immunofluorescence protocol see next section (beginning at step 31).

28. Calculate the amount of virus stock needed to infect a given number of kidney organoids with 10^6^ SARS-CoV-2 infectious particles per organoid (10^6^ PFU per organoid). Keep some of the organoids for the mock control. Prepare 24 organoids for each condition tested, 12 organoids for qRT-PCR and 12 organoids for immunofluorescence.
29. Infect control or diabetic kidney organoids with 10^6^ PFU per organoid.
  a. Use wide orifice 200 μL pipette tips (wo-tips) to place 1 organoid per well of Low-Attachment Surface 96-well plate with 100 μL per well of the Normoglycemic or Hyperglycemic Medium.
    ***Note:*** Use always wo-tips to collect or handle organoids.
    **CRITICAL:** Keep mock and SARS-CoV-2 infected kidney organoids in separate plates to avoid cross-contamination of the mock controls with virus from infected organoids.
  b. Dilute the virus stock into the desired volume of Complete Infection Medium (Virus Working Solution) to have 10^6^ PFU per 100 μL (2 × 10^7^ infectious particles/mL).
  c. Remove the media from each well and add 100 μL of the Virus Working Solution per well of the Low-Attachment Surface 96-well plate containing the organoids. Add the same volume of medium without virus for mock controls.
  d. Incubate the mock or infected organoids at 37°C and 5% CO_2_ for 1 h.
  e. After 1 h incubation, remove the supernatant and dispose as infectious waste.
  f. Wash organoids 3 times with 100 μL of Normoglycemic or Hyperglycemic Medium per well to remove unbound virus. Dispose the supernatants as infectious waste.
    **CRITICAL:** When performing the washing steps pay attention not to aspirate organoids by accident.
  g. Add 100 μL per well of fresh Normoglycemic or Hyperglycemic Medium and incubate the mock or SARS-CoV-2 infected organoids at 37°C and 5% CO_2_ for 1 day.
30. Collection of infected kidney organoids for further analysis at day 1 post-infection (1 dpi).
  a. For each condition tested, use a wo-tip to collect 24 organoids in two 1.5 mL Eppendorf tubes (12 organoids per tube).
  b. Wash the organoids 3 times with 500 μL of PBS 1× per tube to remove unbound virus.
  c. Remove the supernatant and dispose as infectious waste.
  d. For viral RNA detection by qRT-PCR, add 500 μL of Trizol^TM^ to 12 organoids of each condition tested and store the samples at −80°C for further analysis.
  e. For NP detection by immunofluorescence use 4% PFA solution to fix 12 organoids of each condition tested.
    i. In the fume hood, add 500 μL of 4% PFA per Eppendorf tube.
    ii. Incubate the samples for 12–16 h at 4°C.
    iii. In the fume hood remove the fixative and wash the fixed organoids 3 times with 500 μL of PBS 1×.
    iv. Keep the fixed samples in PBS 1× at 4°C for further analysis.

**Pause point:** Fixed organoid specimens are stored at 4°C for further analysis in the following weeks. For longer storage of fixed specimens (up to 6–12 months), we recommend storing them in PBS 1× supplemented with 0.02% of Sodium Azide.

### Immunofluorescence analysis of kidney organoids upon SARS-CoV-2 infection

**Timing: 4–5 days**

**Figure 4:**
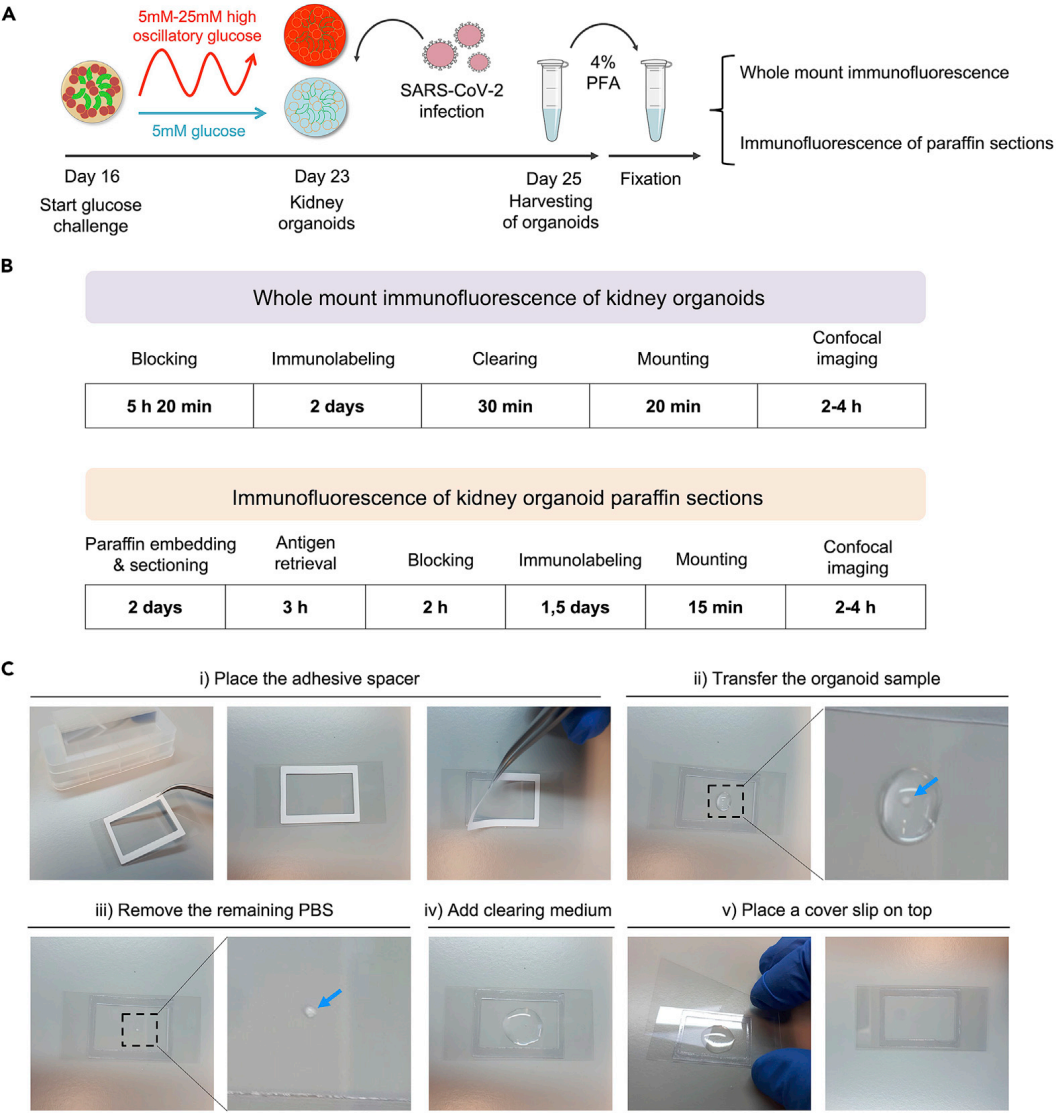
Methodology to analyze kidney organoids by immunofluorescence (A) Timeline of the procedure to emulate a diabetic-like milieu in kidney organoids, infect them with SARS-CoV-2 virus and then harvest and fix them for performing immunofluorescence analysis. (B) Overview of the required steps to perform whole mount immunofluorescence or immunofluorescence in paraffin sections of kidney organoids. (C) Representative photographs to illustrate the procedure for mounting and clearing immunolabeled whole kidney organoid samples for imaging in a confocal microscope.

This section describes the analysis of the extent of SARS-CoV-2 infection in kidney organoids exposed to Control and Diabetic conditions by immunofluorescence. Kidney organoids are analysed at 1 dpi for the detection of NP in combination with *Lotus Tetragonolobus Lectin* (LTL) to detect proximal tubular-like structures and angiotensin-converting enzyme 2 (ACE2). Immunofluorescence is performed in whole mount (see step 32) or by paraffin-sectioning (see step 33) of the organoids (see Figure 4).

31. Divide the number of fixed organoid specimens (previously fixed with 4% PFA; see step 30) to perform either whole mount immunofluorescence or immunofluorescence in paraffin sections.

#### Whole mount immunofluorescence of kidney organoids

**Timing: 4–5 days**

32. For whole mount immunofluorescence of kidney organoids proceed as follows (see Figures 4A and 4B). Troubleshooting 14 and 15.
  ***Note:*** Perform the immunolabelling of at least 3 organoids per experimental condition.
  a. Place 3 organoids per experimental condition in each well of 24-well plate with 400 μL of Tris-Buffered Saline (TBS) 1×. Place 1 additional organoid per experimental condition in individual wells of 24-well plate with 400 μL of 1× TBS for secondary antibody controls. This control is necessary to evaluate the potential non-specific staining of secondary antibodies.
  b. Shake the plate in an orbital shaker at 20°C–22°C for 10 min.
  c. Perform the blocking of the organoids.
    i. Remove the TBS 1× from each well of 24-well plate.
    ii. Add 400 μL of TBS 1× supplemented with 1% Triton X-100 and 6% Donkey Serum (6%DS-Blocking Buffer) per well.
    iii. Incubate for 4 h at 20°C–22°C in an orbital shaker.
  d. Perform an additional blocking step with the Streptavidin/Biotin Blocking Kit, composed by a Streptavidin and a Biotin Solution, to block the organoid endogenous biotin. This step is required when biotinylated LTL is used.
    i. Remove the 6%DS-Blocking Buffer from each well of 24-well plate.
    ii. Wash 1 time with 400 μL of TBS 1× per well for 15 min at 20°C–22°C in an orbital shaker.
    iii. Remove the TBS 1× and add 6–8 drops of the Streptavidin Solution to each well to cover the organoids. Incubate for 20 min at 20°C–22°C under constant shaking.
    iv. Wash the organoids once with 400 μL of TBS 1× per well for 15 min at 20°C–22°C in an orbital shaker.
    v. Remove the TBS 1× from each well and add 6–8 drops of the Biotin Solution to each well to cover the organoids. Incubate for 20 min at 20°C–22°C under constant shaking.
    vi. Wash the organoids once with 400 μL of TBS 1× per well for 15 min at 20°C–22°C in an orbital shaker.
    vii. Remove the TBS 1× from each well and add 400 μL of TBS 1× supplemented with 1% Triton X-100 and 1% Bovine Serum Albumin (BSA) (BSA-1%T-Blocking Buffer)
      **Pause point:** At this stage organoids can be stored in BSA-1%T-Blocking Buffer for 12–16 h at 4°C and start the incubation with primary antibodies the following day.
  e. Prepare the necessary primary antibody solution by diluting the correspondent primary antibodies in BSA-1%T-Blocking Buffer (see materials and equipment section).
    ***Note:*** Calculate spending 250 μL of the primary antibody solution per well of the 24-well plate.
  f. Remove the BSA-1%T-Blocking Buffer from each well and cover the organoids with 250 μL of the primary antibody solution. Incubate for 1 h at 20°C–22°C under constant shaking, then 12–16 h at 4°C and finally at 20°C–22°C for an additional 1 h, under shaking conditions.
    **CRITICAL:** Remember to add 1 organoid per condition as the secondary antibody control. Leave the secondary antibody control organoid in BSA-1%T-Blocking Buffer without the addition of primary antibodies.
    ***Optional:*** Primary antibody incubation at 4°C can be extended up to 24–48 h.
  g. After primary antibody incubation, wash organoids 3 times with 400 μL of BSA-1%T-Blocking Buffer per well for 20 min at 20°C–22°C under shaking conditions.
  h. Prepare the necessary secondary antibody solution by diluting the correspondent secondary antibodies in BSA-1%T-Blocking Buffer (see materials and equipment section).
    ***Note:*** Calculate spending 250 μL of the secondary antibody solution per well of the 24-well plate. Remember to include the secondary antibody control organoid incubating it with the secondary antibody solution.
  i. Centrifuge the secondary antibody solution at 1,200 × *g*, take the supernatant and discard any pellet of precipitates that might have been formed.
    **CRITICAL:** The potential presence of precipitates in the secondary antibody solution could interfere with the imaging of organoids by confocal microscopy.
  j. Remove the BSA-1%T-Blocking Buffer from each well and cover the organoids with 250 μL of the secondary antibody solution per well. Incubate for 4 h at 20°C–22°C in an orbital shaker in darkness. Maintain the darkness conditions until the end of the protocol.
    **CRITICAL:** Prolonged incubation with secondary antibody solution for more than 4 h might cause unspecific antibody adhesion to the organoids.
  k. After secondary antibody incubation, wash the organoids 3 times with 400 μL of TBS 1× per well for 20 min at 20°C–22°C under shaking conditions.
  l. Counterstain the nuclei with DAPI.
    i. Remove the TBS 1× from each well and cover the organoids with 250 μL of the DAPI working solution (dilute the DAPI stock at 1:5000 ratio in TBS 1×).
    ii. Incubate for 12–16 h at 4°C.
    iii. Remove the DAPI solution and add 400 μL of PBS 1× to each well. Keep the immunolabelled organoids at 4°C in the darkness until mounting them for imaging.
      ***Note:*** Avoid storage of immunolabelled organoids longer than 24–48 h.
  m. Perform clearing and mounting of the samples for confocal imaging (See Figure 4C).
    i. Take 1 rectangular cover slip per sample condition and adhere a double-sided sticky iSpacer®.
    ii. Using a wo-tip, transfer 1 immunolabelled organoid, trying to collect the minimum amount of PBS 1×, onto the cover slip. Use a tip to remove as much as possible any residual PBS 1× solution.
      ***Note:*** 3 organoids per experimental condition can be cleared and mounted in the same rectangular cover slip.
    iii. Add a drop of 100 μL of RapiClear® 1.47 clearing reagent to completely immerse the organoid in the clearing solution. Avoid introducing bubbles.
    iv. Incubate for 20 min at 20°C–22°C until the organoid becomes transparent.
    v. Place another rectangular cover slip on top of the cleared organoid and check that the clearing solution spreads and cover the entire surface. The double-sticky iSpacer® allows adhesion of both cover slips.
    vi. Store the mounted organoid samples at 4°C in the darkness until imaging.

#### Immunofluorescence of kidney organoid paraffin sections

**Timing: 4 days**

33. For immunofluorescence of kidney organoid paraffin sections proceed as follows (see Figures 4A and 4B). Troubleshooting 16.
  a. Prepare organoids for paraffin-embedding.
    i. Heat the 0.8% low melting agarose solution at 60°C in a water bath until having a liquid agarose solution. Maintain the agarose solution warm at 37°C.
    ii. Place a layer of 100 μL of 0.8% low melting agarose solution into the bottom of a plastic cryomold. Allow to cool down for 5 min.
    iii. Using a wo-tip, immediately re-suspend the fixed kidney organoids samples (from step 31) in 150 μL of warm 0.8% low melting agarose (37°C) and transfer the organoid–agarose suspension to the agarose-coated plastic cryomold.
      ***Note:*** Place at least 3 fixed kidney organoid specimens per cryomold.
      **CRITICAL:** Ensure that organoids are surrounded by agarose without introducing air bubbles. Place the organoids close to each other.
    iv. Place the prepared molds on ice until the agarose is solidified.
    v. Remove the agarose block containing the organoids from the mold and place it in 1 well of 12-well plate with 1 mL of cold PBS 1×.
      **Pause point:** The agarose blocks with organoids can be stored at 4°C, up to 1 week, for further paraffin embedding. We recommend to store agarose blocks in PBS 1× supplemented with 0.02% of Sodium Azide.
  b. Make paraffin blocks.
    i. Using a spatula, transfer the agarose blocks prepared in step 33a into embedding cassettes.
    ii. Proceed to standard dehydration in an automatic tissue processor (Tissue-Tek® VIP® 6, Sakura or equivalent) following the standard procedure.
    iii. Embed in paraffin at 58°C using metallic molds to obtain paraffin blocks in a paraffin station (Tissue-Tek TEC, Sakura or equivalent).
      **Pause point:** The paraffin blocks can be stored at 20°C–22°C.
  c. Section the paraffin blocks.
    i. Cool down the paraffin blocks on a cold plate (Tissue-Tek, Sakura or equivalent).
    ii. Prepare organoid sections of 3 μm-thickness using a rotary microtome (Leica RM2255 or equivalent).
      **CRITICAL:** Since organoids are quite small and difficult to distinguish within the paraffin block, do not approach the sample by cutting thicker sections to avoid losing the sample.
    iii. Guide the ribbon of paraffin sections into the water bath (Leica HI1210 or equivalent) at 40°C to help unfolding of the sections on the water surface.
    iv. Collect the paraffin sections using charged glass slides. Prepare 20 slides with 2 sections per slide.
      **CRITICAL:** Properly label each slide.
      ***Note:*** We recommend performing serial sectioning.
    v. Dry the slides in an oven at 60°C for 12–16 h.
      **Pause point:** The slides can be stored at 20°C–22°C in a dry place for 6–12 months.
  d. Select the slides for immunofluorescence analysis.
    i. Perform Hematoxylin and Eosin (H&E) staining in slides number 5, 10, 15 (from a total of 20 prepared slides per paraffin block) following standard procedures.
    ii. Observe the H&E-stained slides under the microscope to identify the slides that contain organoid sections.
      ***Note:*** For example, if organoid sections are visible by H&E staining in slides 5 and 10 but not in slide 15, this indicates that organoids sections are majorly present from slide 5 to slide 10.
    iii. Select at least 2 slides per sample to perform immunofluorescence analysis of a given combination of primary antibodies. Take an additional slide for performing the secondary antibody control.
  e. Deparaffinize and re-hydrate the selected slides.
    i. Use an automated tissue processor following the standard procedure.
  f. Perform antigen retrieval.
    i. Immerse the slides in citrate buffer (pH 6).
    ii. Autoclave the immersed slides at 95°C for 20 min.
    iii. Cool down the slides to 20°C–22°C.
      **Pause point:** After antigen retrieval the slides can be stored at 4°C in TBS 1× for 24 h before continuing with the next step.
  g. Perform the blocking of the samples.
    i. Wash the slides with 0.5–1 mL TBS 1× per slide.
    ii. Use 0.5–1 mL of TBS 1× containing 1% Triton X-100 and 3% Donkey Serum (3%DS-Blocking Buffer) to cover the organoid sections in each slide.
    iii. Incubate the samples for 1 h at 20°C–22°C.
      ***Note:*** Use a box for histology slides to make a humidified chamber and horizontally place the slides inside to perform the blocking and the subsequent immunolabelling steps.
  h. Perform an additional blocking step with the Streptavidin/Biotin Blocking Kit as detailed in step 32d. This step is required when biotinylated LTL is used. After that, cover the organoid sections in each slide with 0.5–1 mL of TBS 1× containing 0.5% Triton X-100 and 1% BSA (BSA-0.5%T-Blocking Buffer).
    ***Note:*** Shaking is not required.
  i. Prepare the necessary primary antibody solution by diluting the corresponding primary antibodies in BSA-0.5%T-Blocking Buffer (see materials and equipment section).
    ***Note:*** Calculate spending 200 μL of the primary antibody solution per slide.
  j. Remove the BSA-0.5%T-Blocking Buffer from the slides and use a hydrophobic barrier pen to draw a circle around organoid sections in each slide.
  k. Cover organoid sections in each slide with 200 μL of the primary antibody solution. Incubate the slides for 12–16 h at 4°C.
    **CRITICAL:** Remember to use 1 slide for the secondary antibody control. Leave the secondary antibody control slide in BSA-0.5%T-Blocking Buffer during primary antibody incubation.
    **CRITICAL:** Use a box for histology slides to make a humidified chamber and horizontally place the slides inside. Ensure to place the box containing the slides in a quiet place in the refrigerator to avoid any movement or vibration.
  l. After primary antibody incubation, wash the slides 3 times with 0.5–1 mL of BSA-0.5%T-Blocking Buffer per slide for 5 min at 20°C–22°C.
  m. Prepare the necessary secondary antibody solution by diluting the corresponding secondary antibodies in BSA-0.5%T-Blocking Buffer (see materials and equipment section).
    ***Note:*** Calculate spending 200 μL of the secondary antibody solution per slide.
  n. Cover organoid sections in each slide with 200 μL of the secondary antibody solution. Incubate the slides for 2 h at 20°C–22°C in darkness.
    **CRITICAL:** Ensure to place the box containing the slides in a quiet place to avoid any movement or vibration.
  o. After secondary antibody incubation, wash the slides 3 times with 0.5–1 mL of TBS 1× per slide for 5 min at 20°C–22°C.
  p. Counterstain the nuclei with DAPI for 30 min at 20°C–22°C (see step 32l).
  q. Mount the slides for imaging.
    i. Remove the DAPI solution from the slides.
    ii. Deposit 3–4 drops of Fluoromount-G mounting medium on top of each slide.
      **CRITICAL:** Avoid bubble formation.
    iii. Immediately place rectangular coverslip on top of the slide. Carefully remove the mounting media that may come out from the slide using absorbent paper.
    iv. Seal the borders of the slide with nail polish.
      **Pause point:** The mounted slides can be stored at 4°C in darkness for 1–2 weeks without substantial decrease in fluorescence signal intensity.

34. Acquire images on a confocal microscope. Use the Fiji ImageJ2 version 2.3.0 software for image processing and generation of TIFF files.

***Note:*** Zeiss LSM780 or LSM 880-Airyscan Elyra confocal microscopes were used although other confocal microscopes can be used to obtain similar outcomes. Commonly used acquisition settings would be: scan mode= frame; frame size= 1024 × 1024; line average= 2; bidirectional scanning; speed= 7. A 10× dry objective (Plan-Apochromat 10×/0.3 M27) or a 25× multi-immersion objective (Plan-Apochromat 25×/0.8 Imm Corr DIC M27) can be used to acquire the whole organoid specimen or organoid section by activating the tile scan mode to indicate the borders of the organoid in the x/y direction. The z-stack mode is also activated to define the upper and lower limits of the sample in the z direction. A 40× oil immersion objective (Plan-Apochromat 40×/1.3 Oil DIC M27) can be used for imaging high magnification details of the sample.

## Expected outcomes

Following the procedures detailed here we routinely produce SARS-CoV-2 virus stocks and assess virus stock titers in 95% of plates from which virus production are initiated, obtaining virus stock titers ranging from 10^6^ and 10^7^ PFU/mL.

Additionally we expect the efficient generation of kidney organoids from hPSC in 90% of plates from which differentiations are initiated. Following our methodology kidney organoids develop in free floating conditions using V-bottom 96-well plates in which 1 single organoid forms in each well.

Successful generation of kidney organoids is characterized by the formation of round translucent epithelial structures, so-called RV, visible under the microscope on day 11 of differentiation (Figure 3H), and then progress into nephron-like structures until day 16 of differentiation (Figure 3J). Our procedure leads to the generation of kidney organoids containing segmented nephron-like structures as well as stromal-like cells, endothelial-like cells, among others.^1^^,^^2^^,^^3^^,^^4^

**Figure 5:**
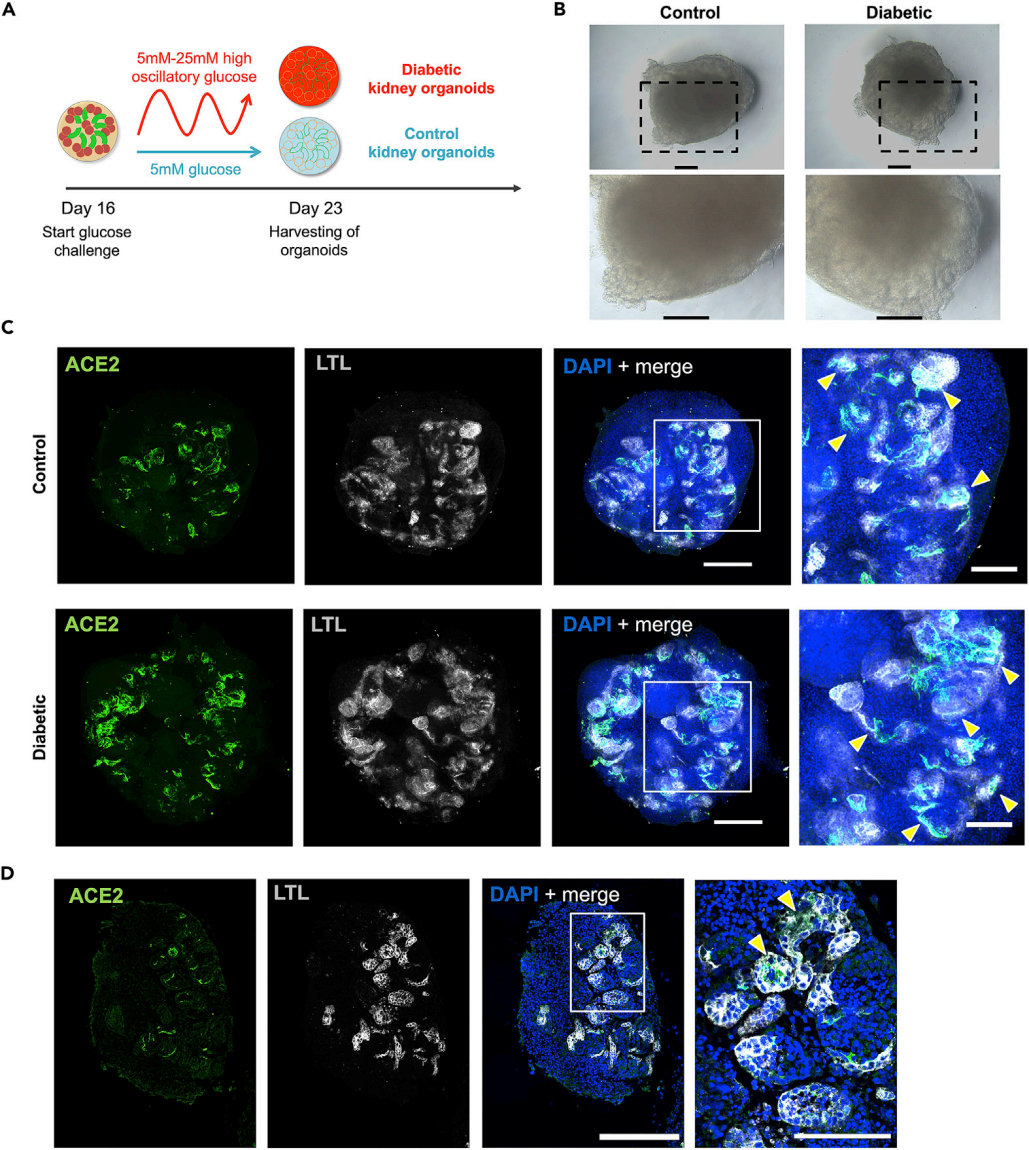
Immunofluorescence analysis of kidney organoids after exposure to diabetogenic-like culture conditions (A) Schematic overview of the procedure to induce control or diabetic kidney organoids. (B) Representative bright field images of control or diabetic kidney organoids. Scale bars, 100 μm. (C) Representative confocal images of control or diabetic kidney organoids analyzed by whole mount immunofluorescence for the detection of ACE2 (green), LTL (gray) and DAPI (blue). Scale bars, 250 μm, 100 μm (high magnification views). (D) Example of representative confocal images that show the detection of ACE2 (green), LTL (gray) and DAPI (blue) by immunofluorescence in an organoid paraffin section. Scale bars, 250 μm, 100 μm (high magnification view). Yellow arrowheads in (C-D) indicate examples of LTL positive proximal tubule-like structures with ACE2 expressing cells.

Recently, we established a high oscillatory glucose regime that leads to early hallmarks of diabetic kidney disease during development and investigated the impact of a diabetic-like milieu on early stages of SARS-CoV-2 infection in hPSC-derived kidney organoids.^1^ Our procedure is highlighted in Figures 5A and 5B. Here, we provide representative confocal images of control and diabetic organoids showing that ACE2 expressing cells (ACE2^+^) are mainly detected within LTL positive proximal tubule-like structures (LTL^+^) (Figures 5C and 5D).^1^

Here we also detail methods for the detection of SARS-CoV-2 infection in control or diabetic kidney organoids taking advantage of whole mount immunofluorescence and immunofluorescence in paraffin sections. In our recent manuscript both immunolabelling techniques have been optimized and validated for the efficient detection of ACE2^+^ and NP expressing cells (NP^+^) along with different renal markers, such as LTL for proximal tubule structures, WT1 for podocyte-like cells, or CD31 for endothelial-like cells.^1^

**Figure 6:**
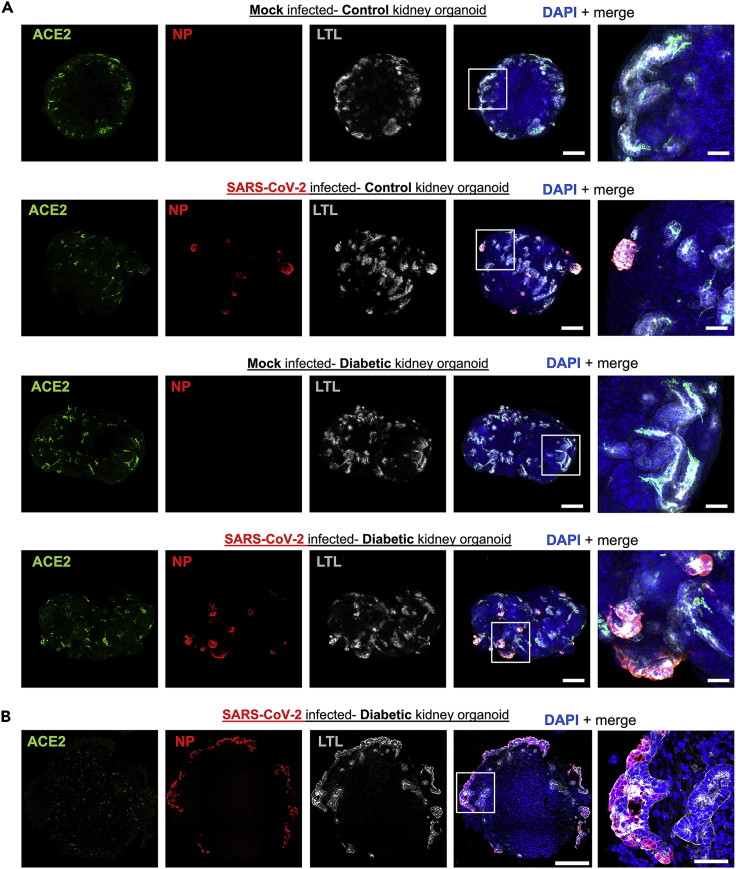
Immunofluorescence analysis of control and diabetic kidney organoids upon SARS-CoV-2 infection (A) Representative confocal images of mock or SARS-CoV-2 infected kidney organoids at 1 dpi for the detection of ACE2 (green), NP (red), LTL (gray) and DAPI (blue) by whole mount immunofluorescence. Scale bars, 200 μm, 50 μm (high magnification views). (B) Example of representative confocal images that show the detection of ACE2 (green), NP (red), LTL (gray) and DAPI (blue) by immunofluorescence in an organoid paraffin section. Scale bars, 200 μm, 50 μm (high magnification view).

Here, we provide the step-by-step procedure for the detection of viral NP together with ACE2 and LTL in mock or SARS-CoV-2 infected kidney organoids under control or diabetic conditions. In Figure 6 we provide representative confocal images of immunolabeled whole mount organoids (Figure 6A) or organoid paraffin sections (Figure 6B) showing that NP^+^ are mainly detected within LTL^+^ in infected organoids.

Overall, the methodology described herein enables side-by-side comparison of SARS-CoV-2 infectivity between control and diabetic kidney organoids. Our read outs allow for the examination of viral NP expression concomitantly with ACE2 and LTL. Further analysis using markers of major kidney compartments can be found in our recent manuscript.^1^

## Limitations

The protocol described herein has been successfully used to study the impact of a diabetogenic-like milieu on early stages of SARS-CoV-2 infection in hPSC-derived kidney organoids.^1^ However, we are aware that *in vitro* models as organoids do not truly recapitulate the full complexity of the human organ. For instance, it should be noted that the procedure described here to generate kidney organoids does not allow for the generation of fully developed vascular networks neither immune cells nor a proper collecting duct system. Nevertheless, our renal differentiation procedure consistently gives rise to kidney organoids containing ACE2^+^ within tubular-like structures,^1^ as well as contain other renal cell populations (i.e., podocyte-like cells, tubule-like cells, endothelial-like cells, among others),^1^^,^^2^^,^^3^^,^^4^ recapitulating in part the multicellular complexity of the native organ and thus providing a valuable *in vitro* model to study SARS-CoV-2 infection in the human context.

In our recent study we have made use of 2 different hPSC lines for the derivation of kidney organoids, including male human embryonic stem cells (ES[4] cell line) and female cord blood iPSCs (CBiPS1sv-4F-40 cell line), confirming infection with SARS-CoV-2 in both genetic backgrounds under non-diabetic and diabetic-like conditions.^1^ It is well known that to model infection successfully it is crucial to ensure the accessibility of the different cell types in the organoid model compared to the native organ.^5^ In this regard, it is important to highlight that ACE2^+^ in kidney organoids are spontaneously exposed and accessible to *in vitro* interventions as SARS-CoV-2 infection. Another important aspect in this regard is that our procedure does not require extracellular matrix (ECM)-related matrices (i.e., Matrigel) for organoid culture and thus viral accessibility is equal between biological replicates. However, as the organoid model system defined here does not recapitulate important features as proper vascularization or immune system, there remains a lack of understanding on complex cell-to-cell interactions and crosstalk upon infection. Further aspects to be studied in the future include exposure time by increasing organoid lifespan and sample analysis upon longer periods post-infection.

## Troubleshooting

### Problem 1

Low cell viability after thawing a cryovial of hPSC on VTN-N-coated plates in Complete E8 Medium. 24 h after thawing hPSC have not attached and most of them appear as floating dead cells in the culture plate (before you begin, step 10; see Figure 1B).

### Potential solution

Ensure that hPSC colonies are at 80% confluency and have the correct morphology (without spontaneous differentiation) before freezing. When freezing hPSC, ensure to gently dissociate hPSC colonies in cell clusters avoiding single cell dissociation and use E8 medium supplemented with 10% DMSO as freezing media. For successful cryopreservation of cells, use a freezing container (i.e., Nalgene® Mr Frosty) to store the cryovials at −80°C. This type of containers ensures a controlled slowdown of the cryovials temperature at 1°C/min cooling rate until reaching −80°C. Keep the cryovials at −80°C for 24 h after which immediately transfer the frozen cryovials to the liquid nitrogen tank. It is also important to thaw quickly the cryovial at 37°C and immediately wash cells to remove the DMSO by adding 10 mL of Complete E8 Medium in a 15 mL falcon tube, centrifuging the cell suspension (5 min, 300 × *g*) and gently re-suspending the cell pellet in fresh Complete E8 Medium. Do not use rock inhibitor.

### Problem 2

Low cell viability after passaging. High cell mortality and too low cell attachment is observed after performing the passage (before you begin, step 13; see Figure 1C).

### Potential solution

Ensure to passage hPSC colonies with the right confluency (80%) and morphology, without spontaneous differentiation. Adjust the timing of exposure to EDTA. Too long EDTA exposure may result in too small cell clusters or single cells, reducing viability. Too short EDTA exposure may result in insufficient cell detachment and exposure of cells to high mechanical stress when trying to detach them, thus reducing viability. After EDTA exposure, use 1 mL of Complete E8 Medium to flush the well and detach de hPSC colonies. They should come out easily. Use another fresh mL of E8 medium to flush again the well and collect the remaining cells. Avoid excessive up and down pipetting of the hPSC clumps suspension. Do not use rock inhibitor to passage the hPSC. Revise that VTN-N coatings have been properly prepared. Avoid using prepared VTN-N coatings that have been stored for more than a week. Remember to routinely check the expiration date and batch of VTN-N and Complete E8 Medium.

### Problem 3

hPSC colonies do not have defined edges, or cells appear less compact within the colonies, or large differentiated cells appear in the culture within or surrounding the hPSC colonies (before you begin, step 13; see Figure 1D).

### Potential solution

Ensure to use freshly prepared VTN-N coatings, Complete E8 Medium and EDTA solution. Ensure to make periodic medium changes to hPSC cultures as recommended. Reduce hPSC colonies density by increasing the splitting dilution ratio. Do not use rock inhibitor for passaging hPSC.

### Problem 4

When plates are revealed with crystal violet staining, plaques are found to be grouped around edges of wells or uneven distributed within wells (step 12 of step-by-step method details).

### Potential solution

Virus inoculum was not well distributed during incubation of virus dilutions for 1 h. Make sure to distribute the virus inoculum across the wells by gently moving the plates from front to back and side to side every 15 min of incubation to allow homogeneous distribution of virus.

### Problem 5

A large area of missing cells or a large plaque-like spot is observed (step 12 of step-by-step method details).

### Potential solution

This could be caused by scratching them when removing the agarose with the spoon. To avoid this, be careful not to touch monolayers with the flat spoon to prevent harming the cell monolayers.

### Problem 6

The number of counted plaques is lower than 5 in all virus dilutions, or the number of counted plaques is bigger than 100 in all virus dilutions (step 13 of step-by-step method details).

### Potential solution

If number of plaques is lower than 5 at all dilutions, this means that virus titer may be less than 1 × 10^2^ PFU/mL. Discard the virus stock and consider making new virus stocks to increase the virus titer. If number of plaques is bigger than 100 at all dilutions, this means that virus titer may be higher than 1 × 10^7^ PFU/mL. In this case, repeat the plaque assay performing further serial dilutions: 10^-6^, 10^-7^ and 10^-8^.

### Problem 7

Low virus titers are obtained (step 13 of step-by-step method details).

### Potential solution

In our hands, most SARS-CoV-2 virus stock titers are between 10^6^ to 10^7^ PFU/mL. With this as reference, some methodological points can be considered to achieve optimal virus titers. (a) Routinely check morphology of Vero-E6 Cells and change to low passage Vero-E6 Cell stocks if any change is appreciated or infectivity has decreased. (b) For proper virus production, infect Vero-E6 Cells at low MOI per cell, usually 0.01. (c) To guarantee that virus supernatants are collected at the optimal time (when virus production peaks), prepare additional flasks to collect the virus supernatants at different time points and check the best titer by the plaque assay.

### Problem 8

24 h after seeding the hPSC in the 24-well plate for differentiation, hPSC appear all together in the middle of the well or uneven distributed across the well, or heterogeneity on cell density is observed among different wells (step 21 of step-by-step method details).

### Potential solution

When plating the hPSC into the 24-well plate, dispense 0.5 mL of the cell suspension per well of the 24-well plate using a p1000-micropipette or a 5 mL-pipette while making sure to homogenize the suspension of cell clumps regularly by gently moving the falcon tube. After plating the cells, manually shake the 24-well plate to allow homogeneous distribution of the cell clumps across the wells, repeat after 30 min. Then, keep the plates in a quite incubator, avoiding any vibration or movement (i.e., opening-closing the incubator) for at least 2 h.

### Problem 9

Cells massively die after CHIR addition (from day 0 to day 3 of renal differentiation), (step 22 of step-by-step method details).

### Potential solution

It is important to take in mind that hPSC seeding density and colony distribution are crucial for achieving efficient renal differentiation and formation of a compact cell monolayer by day 4 of differentiation. If there is excessive cell death upon CHIR stimulation, this might be due to inadequate cell density or inadequate colonies morphology at day 0 of the protocol. For optimal colony density and morphology on day 0 see Figure 3B. If colonies are too small after 24–48 h of plating, replace the media with fresh Complete E8 Medium and wait additional 24 h before starting the differentiation. It is recommendable to optimize the initial cell seeding density for each hPSC line. If above reasons have been excluded, revise that CHIR stocks have been correctly prepared.

### Problem 10

On day 4 of differentiation cells have not formed a confluent and compact cell monolayer as expected at this stage. Instead, areas of loose cells or empty areas are observed in the cultures (see Figure 3D, step 23 of step-by-step method details).

### Potential solution

This could be due to improper hPSC density and colony distribution when starting the differentiation at day 0 of the kidney organoid generation protocol. If this is the case, repeat the experiment using 2 different initial hPSC seeding densities to ensure that 48 h after hPSC seeding, the required confluency and colony distribution is optimal to start the differentiation process. Also, from day 0 to day 4 of the differentiation protocol, take special care when performing media changes since differentiated cells tend to easily detach from the plate. Thus, dispense media slowly to avoid producing excessive pressure against the cells that would damage the cell monolayer and impair the formation of a compact cell monolayer by day 4 of differentiation.

### Problem 11

On day 6 of the kidney organoid generation protocol cell spheroids do not have a compact morphology with defined edges. Instead, cell spheroids appear disaggregated (see Figure 3F, step 24e of step-by-step method details).

### Potential solution

Repeat the experiment and make sure to check the following methodological points. On day 4 of differentiation: (a) when dissociating the cell monolayers into single cells, avoid excessive exposure of the cell monolayer to TrypLE^TM^ Express solution, and completely remove the TrypLE^TM^ Express solution before dissociating the cell monolayer by flushing the cells with media. Do not preform excessive up and down pipetting of the cell suspension since this may harm the cells; (b) after dissociation collect the cell suspension in kidney organoid differentiation medium containing FGF9 and heparin. Do not use medium without FGF9 and heparin supplementation. While counting the cells, keep the cell suspension in the falcon tube at 37°C to preserve cell viability; (c) after cell counting and plating in V-bottom 96-well plates, check that the centrifuge is properly equilibrated when V-bottom 96-well plates are centrifuged to force the aggregation of day 4-differentiated cells into spheroids. After centrifuging the plates, check that a pellet of cells is properly formed at the bottom of each well of the V-bottom 96-well plate. After 48 h of incubation (day 6), each cell pellet should result in the formation of a spheroid.

### Problem 12

On day 11 of the kidney organoid generation protocol RV structures are not visible within organoids under the optical microscope (see Figure 3I, step 24h of step-by-step method details).

### Potential solution

This is due to failed renal differentiation. Repeat the experiment. Check that the initial hPSC density is the optimal for efficient kidney organoid generation (see Figure 3B) and perform media changes regularly as explained in each step of the protocol. Also, revise that the stocks of factors and reagents for medium preparation and basal medium for kidney organoid generation have not expired. Avoid repeated freezing and thawing cycles of the aliquoted stocks and avoid using reconstituted stocks of CHIR, FGF9, Activin A, and heparin for more than 6 months.

### Problem 13

On day 16 of kidney organoid generation nephron-like structures are not observed within organoids under the optical microscope (see Figure 3K, step 25 of step-by-step method details).

### Potential solution

This is due to failed or inefficient renal differentiation. It is possible that the yield of kidney differentiation was low, leading to the formation of other cell types that overgrow within the organoid, thus impairing the development of the few renal structures that could have formed. Repeat the experiment to improve the yield of renal differentiation by following specific recommendations detailed in the protocol herein (i.e., proper initial cell density and colony distribution by day 0, formation of a proper homogeneous and compact cell monolayer by day 4, perform media changes regularly as explained in each step of the protocol, among others). Also, revise that the stocks of factors and reagents for medium preparation and basal medium for kidney organoid generation have not expired. Avoid repeated freezing and thawing cycles of the aliquoted stocks and avoid using reconstituted stocks of CHIR, FGF9, Activin A, and heparin for more than 6 months.

### Problem 14

After performing whole mount immunofluorescence of kidney organoids, the organoid samples used for secondary antibodies control display a high background signal (step 32 of step-by-step method details).

### Potential solution

The secondary antibodies detailed in the protocol herein have been extensively tested and successfully validated for whole mount immunolabelling of kidney organoids with reproducible results. However, make sure to avoid incubating samples with secondary antibodies solution for more than 4 h since this may promote unspecific antibodies binding and cause small antibody precipitates. Also, after secondary antibodies incubation you can perform additional washing steps with increased duration time (i.e., 4 times for 30 min each). Ensure to maintain samples under shaking while preforming the antibodies incubation and washing steps.

### Problem 15

After performing whole mount immunofluorescence of kidney organoids weak signal of immunolabelled proteins is observed across the organoid sample (especially in the middle of the organoid) by confocal microscopy (step 32 of step-by-step method details).

### Potential solution

This could be caused by inefficient antibodies penetration within the organoid samples. The whole mount immunofluorescence protocol detailed herein has been optimized for the efficient immunolabelling of kidney organoids. Ensure to maintain samples under shaking while preforming the antibodies incubation and washing steps. Primary antibodies incubation step can be extended up to 48 h.

### Problem 16

After performing immunofluorescence of kidney organoid paraffin sections, uneven immunolabelling of organoid sections is observed by confocal microscopy (step 33 of step-by-step method details).

### Potential solution

This could be caused by heterogeneous exposure of the antibody solution in the sample. Ensure that there has not been any problem during de-paraffinization and re-hydration of organoid paraffin sections. Ensure that organoid sections do not dry during any of the immunolabelling steps, especially during primary antibodies incubation.

## Resource availability

### Lead contact

Further information and requests for resources and reagents should be directed to and will be fulfilled by the lead contact, Nuria Montserrat (nmontserrat@ibecbarcelona.eu).

### Materials availability

This study did not generate new unique reagents.

## Data Availability

The published article includes all datasets generated or analyzed during this study.
